# Factors Associated With MALDI-TOF Mass Spectral Quality of Species Identification in Clinical Routine Diagnostics

**DOI:** 10.3389/fcimb.2021.646648

**Published:** 2021-03-16

**Authors:** Aline Cuénod, Frédéric Foucault, Valentin Pflüger, Adrian Egli

**Affiliations:** ^1^ Applied Microbiology Research, Department of Biomedicine, University of Basel, Basel, Switzerland; ^2^ Division of Clinical Bacteriology and Mycology, University Hospital Basel, Basel, Switzerland; ^3^ Mabritec AG, Riehen, Switzerland

**Keywords:** MALDI-TOF mass spectrometry, quality control, standardisation, species identification, microbial diagnostics

## Abstract

**Background:**

An accurate and timely identification of bacterial species is critical in clinical diagnostics. Species identification allows a potential first adaptation of empiric antibiotic treatments before the resistance profile is available. Matrix assisted Laser Desorption Ionization Time of Flight mass spectrometry (MALDI-TOF MS) is a widely used method for bacterial species identification. However, important challenges in species identification remain. These arise from (i) incomplete databases, (ii) close relatedness of species of interest, and (iii) spectral quality, which is currently vaguely defined.

**Methods:**

We selected 47 clinically relevant bacterial isolates from 39 species, which can be challenging to identify by MALDI-TOF MS. We measured these isolates under various analytical conditions on two MALDI-TOF MS systems. First, we identified spectral features, which were associated with correct species identification in three different databases. Considering these features, we then systematically compared spectra produced with three different sample preparation protocols. In addition, we varied quantities of bacterial colony material applied and bacterial colony age.

**Results:**

We identified (i) the number of ribosomal marker peaks detected, (ii) the median relative intensity of ribosomal marker peaks, (iii) the sum of the intensity of all detected peaks, (iv) a high measurement precision, and (v) reproducibility of peaks to act as good proxies of spectral quality. We found that using formic acid, measuring bacterial colonies at a young age, and frequently calibrating the MALDI-TOF MS device increase mass spectral quality. We further observed significant differences in spectral quality between different bacterial taxa and optimal measurement conditions vary per taxon.

**Conclusion:**

We identified and applied quality measures for MALDI-TOF MS and optimized spectral quality in routine settings. Phylogenetic marker peaks can be reproducibly detected and provide an increased resolution and the ability to distinguish between challenging species such as those within the *Enterobacter cloacae* complex, *Burkholderia cepacia* complex, or viridans streptococci.

## Introduction

Matrix assisted Laser Desorption Ionization Time of Flight mass spectrometry (MALDI-TOF MS) has revolutionised microbial diagnostics. Due to its minimal hands-on and turnaround time, low costs, and high accuracy it has become the method of choice for bacterial species identification in clinical diagnostics (Angeletti and Ciccozzi, 2019; Rodríguez-Sánchez et al., 2019). Multiple studies have highlighted the potential of MALDI-TOF MS to identify virulent or resistant bacterial sub-lineages within a species (Wolters et al., 2011; Christner et al., 2014). Despite these potential applications, important challenges remain for routine diagnostics, such as the inability to properly differentiate clinically relevant taxonomic groups, such as the species within the *Burkholderia cepacia* complex (Fehlberg et al., 2013), the *K. pneumoniae* complex (Dinkelacker et al., 2018) or viridans *s*treptococci (Angeletti et al., 2015). Challenges in species identification arise from (i) incomplete databases, (ii) close relatedness of the bacterial species of interest, and (iii) poor spectral quality.

Species identification through commonly used MALDI-TOF MS systems is based on the comparison of unknown spectra to reference spectra databases through pattern matching. MALDI-TOF mass spectra consist of peaks from highly abundant, intracellular proteins including ribosomal subunit proteins, which are present in high copy numbers in replicating bacterial cells (Fenselau and Demirev, 2001; Ryzhov and Fenselau, 2001). With the abundance of bacterial whole genome sequences, reference databases comprising predicted ribosomal subunit masses have become an alternative to pattern based microbial identification in MALDI-TOF MS. The mass of ribosomal subunits can be directly calculated from genomic sequences, as they are relatively conserved and rarely post-translationally modified. Their potential to serve as MALDI-TOF MS biomarkers has been applied to clinically relevant phylogenetic groups (Hotta et al., 2010; Ziegler et al., 2015; Rothen et al., 2019), and multiple databases using marker masses predicted from genomic data are now available (Kassim et al., 2017; Ojima-Kato et al., 2017; Tomachewski et al., 2018). A ribosomal marker based approach has successfully been applied to distinguish between subspecies and clonal complexes within species such as *Streptococcus agalactiae* and *Escherichia coli* (Matsumura et al., 2014; Lafolie et al., 2015; Rothen et al., 2019).

When MALDI-TOF MS was first applied for microbial species identification (Anhalt and Fenselau, 1975) and in its first years in routine diagnostics, samples were processed using a protein extraction protocol (Patel, 2015). However, as high accuracies in species identification have been reported using a much simpler procedure, applying bacterial colonies directly onto the MALDI-TOF MS target plate has become the standard sample preparation protocol in routine diagnostics (Bizzini et al., 2010; van Veen et al., 2010). Although the Clinical and Laboratory Standard Institute (Pennsylvania, USA) has published a guideline on bacterial identification by MALDI-TOF MS (Branda et al., 2017), the definition criteria of spectral quality remain vague. Many diagnostic laboratories have developed their own Standard Operating Procedures for sample preparation and interpretation of species identification by MALDI-TOF MS. Currently it is already well established that MALDI-TOF mass spectral quality is influenced by the amount of bacterial colony material added to the target plate, the age of the bacterial colony, as well as the sample preparation protocol used (Alatoom et al., 2011; Croxatto et al., 2012; Veloo et al., 2014). However, there is a clear lack of an optimal and standardised sample preparation and data analysis workflow. Criteria defining the spectral quality will help to compare differences in preparation and analytical workflows. Closing this gap will substantially increase the reproducible detection of phylogenetic marker peaks in MALDI-TOF mass spectra acquired and thereby improve species identification in routine diagnostics.

The purpose of this study is to (i) identify quantitative spectral features suitable to define spectral quality, (ii) compare the influence of sample preparation protocols for bacterial identification by MALDI-TOF MS, and (iii) raise awareness for the potential of an increased resolution of MALDI-TOF MS for subtyping and associated limitations.

We have selected 47 clinically relevant bacterial isolates from 39 species and measured these under various conditions on two different MALDI-TOF MS systems. First, we identified spectral features, which positively correlate with correct species identification. Considering these, we systematically compared spectral quality produced with different sample protocols, with varying amounts of bacterial colony material applied, and with varying bacterial colony age.

## Materials and Methods

### Bacterial Isolates

We selected 47 clinically relevant bacterial isolates from public and in-house strain collections. The included 39 species can be challenging to identify using MALDI-TOF MS, either because intracellular proteins cannot be ionised easily due to cell wall composition (e.g. *Corynebacterium* spp.), or because of their close relatedness to another bacterial species (e.g. *Klebsiella oxytoca/Klebsiella michiganensis*; *Shigella/Escherichia coli*).

The bacterial isolates were assigned to 8 phylogenetic groups (**Table 1**). For the strains in each group, we expect both comparable spectral features (e.g. total number of peaks detected) and lysis characteristics, respectively. For the evaluation of species identification, the group ‘*Streptococcus*’ was further split up into ‘viridans streptococci’ and ‘other streptococci’ as the former group are of special interest in clinical routine diagnostics.

**Table 1:** Strains included in this study.

#	Species	Strain collection	Internal strain number	Group	NCBI/ENA Accession Number
01	*Klebsiella pneumoniae*	in-house	602149-19	*Enterobacteriaceae*	SAMN16951201
02	*Klebsiella oxytoca*	in-house	708776-17	*Enterobacteriaceae*	SAMN12212273
03	*Klebsiella grimontii*	in-house	132656-17	*Enterobacteriaceae*	SAMN12212117
04	*Klebsiella michiganensis*	in-house	401065-17	*Enterobacteriaceae*	SAMN12212153
05	*Klebsiella aerogenes*	in-house	717657-17	*Enterobacteriaceae*	SAMN12212322
06	*Klebsiella variicola*	in-house	717892-17	*Enterobacteriaceae*	SAMN12212293
09	*Escherichia coli*	in-house	807627-2-16	*Enterobacteriaceae*	SAMN16951202
10	*Escherichia coli*	in-house	807628-3-16	*Enterobacteriaceae*	SAMN16951203
11	*Escherichia coli*	in-house	804255-13	*Enterobacteriaceae*	SAMN16951204
12	*Escherichia coli*	in-house	805237-12	*Enterobacteriaceae*	SAMN16951205
13	*Shigella flexneri*	in-house	300666-18	*Enterobacteriaceae*	SAMN16951206
14	*Shigella flexneri*	in-house	301552-18	*Enterobacteriaceae*	SAMN16951207
15	*Shigella sonnei*	commercial	DSMZ-5570	*Enterobacteriaceae*	SAMN16951208
16	*Shigella sonnei*	in-house	301974-17	*Enterobacteriaceae*	SAMEA104430192
35	*Enterobacter sichuanensis*	in-house	403902-15	*Enterobacteriaceae*	SAMN16951209
36	*Enterobacter hormaechei*	commercial	ATCC-49162	*Enterobacteriaceae*	SAMN16951210
37	*Enterobacter asburiae*	commercial	ATCC-35956	*Enterobacteriaceae*	SAMN16951211
38	*Enterobacter ludwigii*	commercial	DSMZ-15213	*Enterobacteriaceae*	SAMN16951212
07	*Listeria monocytogenes*	in-house	107373-13	*Listeria*	SAMN16951213
08	*Listeria monocytogenes*	in-house	O1910-17	*Listeria*	SAMN16951214
17	*Burkholderia cepacia*	in-house	208050-16	*Burkholderia*	SAMN16951215
18	*Burkholderia contaminans*	in-house	O-13	*Burkholderia*	SAMEA54114418
19	*Burkholderia multivorans*	in-house	O-10	*Burkholderia*	SAMEA54118168
20	*Burkholderia cenocepacia*	in-house	O-3	*Burkholderia*	SAMEA54110668
21	*Bordetella bronchiseptica*	in-house	502474-16	*Bordetella*	SAMN16951216
22	*Bordetella pertussis*	commercial	ATCC-9797	*Bordetella*	SAMN16951217
23	*Bordetella parapertussis*	commercial	ATCC-53893	*Bordetella*	SAMN16951218
24	*Streptococcus pneumoniae*	in-house	144265-17	*Streptococcus*	SAMN16951219
25	*Streptococcus infantis*	in-house	131226-17	*Streptococcus*	SAMN16951220
26	*Streptococcus gordonii*	commercial	ATCC-33399	*Streptococcus*	SAMN16951221
27	*Streptococcus gallolyticus*	in-house	PRA0000041	*Streptococcus*	SAMN16951222
28	*Streptococcus lutetiensis*	commercial	DSMZ-15350-TS	*Streptococcus*	SAMN16951223
29	*Streptococcus pseudopneumoniae*	in-house	610886-17	*Streptococcus*	SAMN16951224
30	*Streptococcus equinus*	commercial	ATCC-9812	*Streptococcus*	SAMN16951225
31	*Streptococcus dysgalactiae*	in-house	STO0000159	*Streptococcus*	SAMN16951226
32	*Streptococcus dysgalactiae*	in-house	602125-13	*Streptococcus*	SAMN16951227
39	*Staphylococcus aureus*	in-house	351358-18	*Staphylococcus*	SAMN16951228
40	*Staphylococcus schweitzeri*	commercial	DSM-28300-TS	*Staphylococcus*	SAMN16951229
41	*Staphylococcus argenteus*	commercial	DSMZ-28299-TS	*Staphylococcus*	SAMN16951230
42	*Corynebacterium amycolatum*	commercial	ATCC-700206	*Actinobacteria*	SAMN16951231
43	*Corynebacterium urealyticum*	commercial	DSMZ-7109	*Actinobacteria*	SAMN16951232
44	*Gardnerella vaginalis*	commercial	ATCC-14018	*Actinobacteria*	SAMN16951233
45	*Winkia neuii*	in-house	STO0000012	*Actinobacteria*	SAMN16951234
46	*Actinomyces israelii*	commercial	ATCC-10048	*Actinobacteria*	SAMN16951235
47	*Pasteurella multocida*	commercial	ATCC-11039	Gram negative Anaerobes	SAMN16951236
33	*Bacteroides fragilis*	in-house	609216-11	Gram negative Anaerobes	SAMN16951237
34	*Bacteroides fragilis*	in-house	600609-16	Gram negative Anaerobes	SAMN16951238

Strains were either retrieved from in-house or commercial strain collections. Strains which are assigned the same ‘Group’ are expected to respond similarly to varying sample protocols, quantities of bacterial colony material applied and varying bacterial colony age.

### Whole Genome Sequencing

Isolates were grown on Columbia 5% Sheep Blood Agar (bioMérieux, Marcy-l’Étoile, France) and DNA was extracted using the QIACube with the QIAamp DNA Mini Kit (QIAGEN, Hilden, Germany). After quality control of the DNA by Tapestation (Agilent, Santa Clara, USA), tagmentation libraries were generated as described by the manufacturer (Nextera Flex kit, Illumina, San Diego, USA). The genomes were sequenced under 24x multiplexing using a paired end 150 base pairs V3 reaction kit on an Illumina NextSeq500 instrument (Illumina) reaching an average coverage of approximately 60-fold for all isolates. The resulting raw reads were and assembled using Spades (v3.13) (Bankevich et al., 2012) *via* Unicycler (v0.3.0b) (Wick et al., 2017) using default settings. All accession numbers can be found in **Table 1**. Species identification of all strains was performed by comparing genomic sequences to bacterial type strains using Average Nucleotide Identity (ANIm) (Richter and Rosselló-Móra, 2009) and *via* the TrueBac ID database (Ha et al., 2019). For strains of the genus Bordetella we used ribosomal Multi-Locus Sequence Typing for additional confirmation of the species identity (Jolley et al., 2012).

### *In Silico* Prediction of Ribosomal Subunit Protein Masses From WGS Data

The molecular weight of 56 ribosomal subunits were predicted as previously described (Ziegler et al., 2015; Rothen et al., 2019). Briefly, tblastn (v 2.2.31+) was used to extract the amino acid sequences of 56 ribosomal subunits from whole genome assemblies. The most frequent post translational modifications (Arnold and Reilly, 1999), specifically N-terminal methionine loss (Frottin et al., 2006) and methylation, were considered for subsequent prediction of the monoisotopic molecular weights of the ribosomal subunit proteins. For the ribosomal subunit protein L33, we added 15 Daltons to the predicted molecular weight for the genera *Enterobacter*, *Escherichia*, *Shigella*, *Klebsiella* and *Pasteurella*, accounting for a single methylation of these proteins.

### Spectra Quality Variables

All scripts used in the course of this study can be accessed *via* GitHub (https://github.com/appliedmicrobiologyresearch/MALDI-TOF-mass-spectral-quality-study).

We queried each spectrum for the following features: (i) number of peaks, (ii) peak with the highest m/z value, (iii) m/z value of the peak at the 90^th^ percentile, (iv) fraction of peaks with a m/z value > 10,000, and (v) sum of the intensity of all detected peaks. As the highest peak often corresponds to technical artefacts, we included the m/z value of the peak at the 90^th^ percentile for further analysis.

Furthermore, we queried each spectrum for the presence and intensity of ribosomal marker peaks predicted from the genomic sequence of the respective strain using an 800 ppm error range. If multiple peaks were detected in this error range, the one with the highest intensity and lower measurement error was considered for further analysis. Bacterial strains encode variable number of ribosomal markers in the mass range of 2,000–20,000 Da. We therefore normalised the number of detected marker peaks by dividing through the number of predicted ribosomal marker peaks in the MALDI-TOF MS mass range, when comparing between the bacterial taxa.

To quantify the measurement error, we calculated the mean distance between predicted and detected m/z value of ribosomal marker peaks for each spectrum. In order to estimate reproducibility, we calculated the ‘fraction of reproducibly detected peaks’. We defined this as the number of peaks, which were detected in at least three out of four technical replicates using a bin size of 800 ppm, divided by the number of peaks in each spectrum. A more detailed and graphical explanation of the MALDI-TOF mass spectral features analysed in this study can be found in **Supplementary Methods 1**.

Further, we evaluate which of the above MALDI-TOF mass spectral features are good proxies for spectra quality and are associated with a correct species identification. We compared spectra for which the correct species was identified to spectra where the correct species could not be identified. Spectra for which the correct species could not be identified included spectra with wrong species being identified and spectra for which no species identification was possible. Henceforward, we will refer to these collectively as ‘incorrectly identified spectra’. We performed this analysis exclusively on species which are covered by all three databases included in this study (**Supplementary Table S1**) and excluded empty spectra.

In order to assess how spectra quality impacts species identification accuracy, we included spectra acquired using the ‘direct smear’, the ‘25% formic acid (FA) overlay’ or the ‘simple protein extraction’ method (see section ‘Variation of sample preparation’ for details) of the *Enterobacter cloacae complex* (*Enterobacter hormaechei*, *Enterobacter asburiae* and *Enterobacter ludwigii*), the *Burkholderia cepacia complex* (*Burkholderia contaminans*, *Burkholderia multivorans* and *Burkholderia cenocepacia*), and viridans streptococci (*Streptococcus pneumoniae* and *Streptococcus pseudopneumoniae*). We assigned these spectra to three intensity levels, by dividing the sum of the intensities of all detected peaks in three equal parts per group.

### MALDI-TOF MS Spectra Acquisition

All MALDI-TOF mass spectra acquired for this study can be accessed *via* the Open Science Foundation (https://osf.io/ksz7r/). The bacterial isolates were cultured from Microbank™ freezing beads (Pro-lab Diagnostics, Toronto, Canada) onto 5% Sheep Blood agar plates (bioMérieux, Marcy-l’Étoile, France) and subcultured before MALDI-TOF mass spectra acquisition. Strains were incubated under aerobic conditions at 37°C except for strains of the species *Bacterioides fragilis, Actinomyces israelii*, and *Winkia neuii*, which were incubated under anaerobic conditions using a whitley A95 anaerobic workstation (Don Whitley Scientific Limited, Bingley, United Kingdom). Strains of the species *Streptococcus pneumoniae*, *Bordetella pertussis*, and *Bordetella parapertussis* were incubated under 5% enriched CO_2_ conditions. All mass spectra were acquired on reusable steel target plates [MBT Biotarget 96 (Bruker Daltonics, Bremen, Germany) and steel target plates (Industrietechnik mab AG, Basel, Switzerland)].

### Variation of Sample Preparation

We cultured the bacterial strains as described above. We prepared the strains under three different short protocols, all of which are frequently used in microbial diagnostics: (i) ‘Direct smear’ method: using a plastic inoculation needle, we transferred bacterial colonies onto a steel target plate and overlaid each spot with 1 µl α-Cyano-4-hydroxycinnamic (CHAC) matrix (Sigma-Aldrich, St. Louis, USA) and left it to air dry completely before MALDI-TOF MS measurements. (ii) ‘25% FA overlay’: using a plastic inoculation needle, we transferred bacterial colonies onto a steel target plate and overlaid each spot with 1 µl of 25% formic acid (Sigma-Aldrich, St. Louis, USA) and left it to air dry completely before applying 1 µl of CHAC matrix onto each spot. The target plates were left to air dry completely before MALDI-TOF MS measurements. (iii) ‘Simple protein extraction’: we transferred a heaped 1 µl inoculation loop of bacterial colony material into 1 ml PBS, rigorously vortexed, and centrifuged for 5 min at 17,000 x g. We removed the supernatant and added 30 µl 70% formic acid and dissolved the pellet by pipetting up and down. 30 µl acetonitrile (Sigma-Aldrich, St. Louis, USA) were added and the mixture was vortexed before centrifuging for 5 minutes at 17,000 x g. Next, 5 µl of the supernatant were mixed with 25 µl of CHAC matrix before spotting onto the steel target plate.

We performed measurements as quadruplicate on a Bruker microflex LT/LS ‘smart’ (Bruker Daltonics, Bremen, Germany) and a Shimadzu Axima Confidence (Shimadzu, Kyoto, Japan) MALDI-TOF MS device as technical replicates and repeated on three different days with fresh subcultures as biological replicates.

### Variation of Bacterial Colony Age

We grew the strains over 1, 2, 3, 4, 5 or 6 nights before preparing them for measurement using the ‘25% FA overlay’ method described above. Each overnight culture corresponds to 18 - 24 hours of incubation time. We performed measurements as quadruplicate on a Bruker microflex LT/LS ‘smart’ and a Shimadzu Axima Confidence MALDI-TOF MS device.

### Variation of Bacterial Colony Material Quantity

The amount of bacteria transferred onto the MALDI-TOF MS steel target plate has been shown to impact spectral quality (Branda et al., 2017). The direct transfer of bacteria onto a steel target plate is difficult to standardise. We therefore decided to measure bacterial suspensions at different dilutions to assess the impact of the amount of bacterial colony material measured on mass spectral quality. We randomly selected the following two strains per phylogenetic group for this experiment: *Enterobacteriaceae:* #07, #08*; Listeria:* #09, #10; *Burkholderia:* #17, #19; *Bordetella:* #21, #23; *Streptococcus:* #26, #27; *Staphylococcus:* #39, #40; *Actinobacteria*: #45, #46; Gram negative anaerobes: #33, #34.

We transferred a heaped 1 µl inoculation loop of bacterial colony material into 200 µl of TMA (1x) buffer (Sigma-Aldrich, St. Louis, USA). Next, 5 µl of the bacterial mixture was diluted in 25 µl of CHAC matrix (1:5 dilution) and spotted onto the target plates. 5 µl of the suspension were transferred into a new tube containing 25 µl CHAC matrix (1:25 dilution). We continued the serial dilution up to a factor of 1:15,625. As the majority of measurements with 1:3,125 and 1:15,625 dilutions yielded empty spectra, these were excluded from further analysis. We measured quadruplicates on two MALDI-TOF MS devices as technical replicates and repeated on three different days as biological replicates.

### Variation of Time After Calibration to Assess the Impact on Measurement Precision

We performed the measurements on two microflex biotyper devices (LH/LS and LH/LS ‘smart’). Both devices were calibrated using the Bacterial Test Standard (BTS, Part.-Nr. 8255343, Bruker Daltonics, Bremen, Germany) and steel target plates (Bruker Daltonics, Bremen, Germany).

We used an *E. coli* strain from our strain collection (*E. coli* 805237-12) for these measurements as strains of this species generally yield rich spectra using routine sample preparation. We transferred bacteria onto a steel target plate, overlaid with 1 µl 70% FA, left to air dry completely before applying 1 µl CHAC matrix. Each measurement was performed in quadruplicate on two different target plates and MALDI-TOF MS devices as technical replicates and repeated by picking three different colonies as biological replicates. Spectra were acquired on days 1-7 after calibration on the same target plate which was used for calibration. BTS was measured on the row A of the target plate, measurements on day 1-7 after calibration were measured on rows B, C, D, E, F, G and H, respectively. Both MALDI-TOF MS devices where used for microbial species identification in routine diagnostics over the duration of this experiment with a median of 39 (Interquartile range (IQR): [32, 51]) and 137 (IQR: [123, 173]) of routine measurements per day on the microflex biotyper LH/LS and the microflex biotyper LH/LS ‘smart’, respectively.

### MALDI-TOF MS Spectra Processing

In order to be most comparable to spectra acquired and processed in microbial routine diagnostic, we picked the peaks using default settings by the softwares included in the microflex Biotyper or the Axima Confidence system. Spectra acquired on microflex Biotyper devices were exported as ‘fid’ files and peak picking was performed in the flexAnalyses software (v3.4) and exported as ‘.txt’ files. Spectra acquired on the Axima Confidence devices were exported as ‘.mzXml’ files. These do already exclusively contain m/z values and intensities of picked peaks and were converted to ‘.txt’ files. We subsequently exclusively worked with the intensity and m/z value of these picked peaks, and did not consider further peak characteristics such as the resolution or the signal to noise ratio of a peak.

We excluded spectra as contaminations for which the identified genus did not match the genus identified by ANIm. Strain 17 and strain 20 are missing in one out of three repetitions of the ‘simple protein extraction’ protocol, strain 32 is missing from day 6 of the and of strain 46 only three technical replicates were acquired on the Axima Confidence device using the ‘direct smear’ method.

### Species Identification

Each spectrum acquired on a Bruker device was compared to the MALDI Biotyper database (MALDI Biotyper Compass Library, Revision E (Vers. 8.0, 7854 MSP, RUO)) included in the flexControl Software v3 (Bruker Daltonics, Bremen, Germany). Spectra acquired on the Axima Confidence device were analyzed with the VitekMS database (bioMérieux, Marcy-l’Étoile, France) (v3.2). Furthermore, we compared each spectrum to a ribosomal marker based database (PAPMID™ (Kassim et al., 2017), Mabritec AG, Riehen, Switzerland). In this study, we used this marker based approach as a subtyping module and each spectrum was compared only to a subset of bacterial species. Spectra of the species *Escherichia coli*, *Shigella flexneri*, *Shigella sonnei*, *Streptococcus gordonii*, *Streptococcus gallolyticus*, *Streptococcus lutetiensis*, *Streptococcus equinus*, *Streptococcus dysgalactiae, Corynebacterium amycolatum, Corynebacterium urealyticum, Gardnerella vaginalis, Winkia neuii, Actinomyces israelii, Pasteurella multocida*, and *Bacteroides fragilis* were compared to databases including mass profiles of the respective bacterial family. Spectra of the closely related *Klebsiella* spp., *Enterobacter cloacae complex, Listeria* spp.*, Burkholderia cepacia complex, Bordetella* spp.*, Staphylococcus aureus complex*, and viridans streptococci were identified using marker based subtyping modules exclusively including the species of the respective phylogenetic complex. Henceforward, species identification by these subtyping modules and using PAPMID™ database, both based on the detection of ribosomal marker peaks will be referred to as PAPMID™.

The MALDI Biotyper system classifies species identification according to log scores: mass spectra yielding a log score above 2.0 are assigned the label ‘highly confidence identification’, whereas spectra with a log score between 1.7 and 2.0 are assigned ‘low confidence identification’. Spectra with a log score lower than 1.7 are assigned ‘no organism identification possible’. For each spectrum with a log score above 1.7, we evaluated whether the species assigned by the MALDI Biotyper database corresponds to the true species identity determined by whole genome sequence analysis and ANIm. For species with a log score ≥ 2.0 we furthermore evaluated, how many species were assigned a log score ≥2.0.

Similar to the MALDI Biotyper database, the VitekMS database assigns scores to each species classification. Furthermore, each species identification is assigned a *Confidence level* [%] and a *Type of identification*, which is either ‘Single Choice’ or ‘Low Discrimination’ and indicates whether the species identification was unambiguous or whether the database could not unambiguously discriminate between two or more species entries. Identifications with an assigned probability, lower than a probability threshold (60%) are not assigned an unambiguous species label. In this situation, due to low confidence values, the Type of identification ‘No Identification’ or ‘Low Discrimination’ is assigned. For spectra with a Type of identification other than ‘No Identification’, we evaluated the Type of Identification and whether the assigned species corresponds to the true species identity of the measured strains.

We compared all spectra in our dataset to a ribosomal marker based database (PAPMID™). Marker based species identification tools such as the PAPMID™ database assign scores which correspond to the number of ribosomal marker peaks detected. The bacterial species is assigned for which most marker masses could be detected in a mass spectrum.

If a spectrum matches a maximal number of marker masses for multiple profiles of the same species, an unambiguous single species identification is assigned. If a spectrum matches an equal maximal number of marker masses from different species, multiple species are assigned (‘multi-species Identification’). Species identifications with fewer marker peaks detected than the taxon-specific identification threshold, are assigned the label ‘No identification possible’. The taxon specific thresholds used in this study were 20 for the species of the *Enterobacter cloacae complex*, 15 for *Klebsiella* spp. and *Escherichia coli/Shigella*, 7 for the species within the *S. aureus* complex, and 10 for all other phylogenetic groups included in this study.

### Statistical Analysis

We used paired Wilcoxon rank tests when comparing spectra acquired from the same strains under different conditions. We excluded spectra of strains which were missing in one of the sets of interest. We used unpaired wilcoxon rank tests when comparing spectra acquired from different strains, e.g. when comparing between different phylogenetic groups or between correctly and incorrectly identified spectra.

When reporting comparisons in the running text, we refer to spectra acquired on the microflex Biotyper if not explicitly stated otherwise and use the nomenclature ‘median (lower bound of the IQR, upper bound of the IQR)’, throughout the study. Results and summary plots for spectra acquired on the Axima Confidence system can be found in the supplement.

We report the exact p-values when these are > 0.0001 and report use ‘****’ for p-values < 0.0001. All analysis was performed in R (4.0.3) using the ggpubr (4.0) package.

## Results

### Defining MALDI-TOF Mass Spectral Quality

In order to investigate spectral quality of the different datasets, we first assessed which spectral features are associated with a correct species identification with all databases and therefore suitable as quantitative measures for spectral quality. The spectra analysed here include a range of mass spectral quality, and were acquired using all different sample preparation protocols examined in this study and for the species included in three databases (MALDI Biotyper, VitekMS, PAPMID™) (**Supplementary Table S1**).

**Figure 1:**
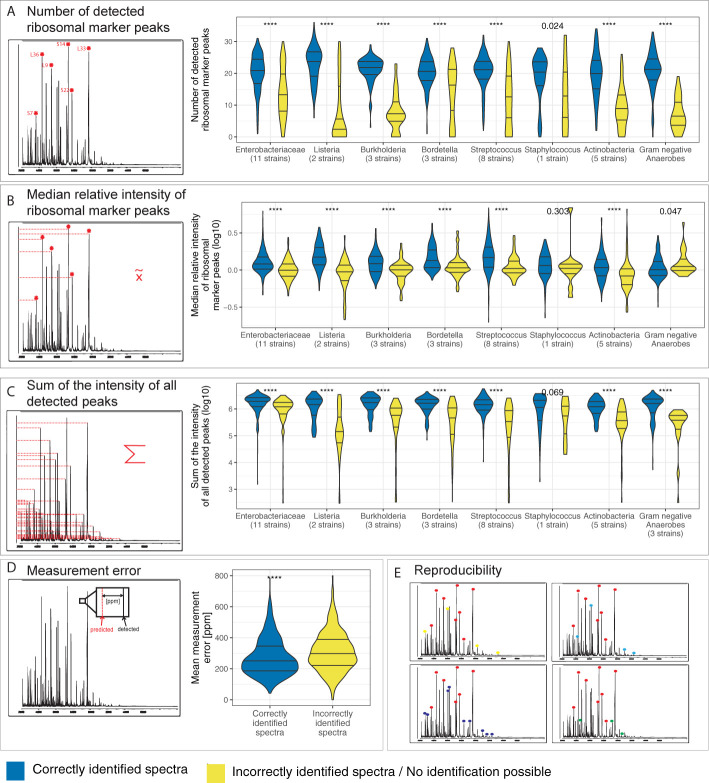
Spectra features compared between correctly and incorrectly identified spectra per phylogenetic group. Species identification was performed by the MALDI Biotyper database and spectra were acquired on a microflex Biotyper. **(A)** the number of detected ribosomal marker peaks, **(B)** the median relative intensity of these, **(C)** the sum of intensity of all detected peaks, **(D)** measurement error and **(E)** reproducibility i.e. number of peaks detected in three out of four technical replicates divided by the total number of peaks in a spectrum. ****p-value < 0.0001.

Five spectral features are good proxies for the correct species identification. In correctly identified spectra (i.e. high spectral quality) over all phylogenetic groups we found an increase in the number of ribosomal marker peaks detected (median = 22 IQR = (18, 25) (same nomenclature used throughout the paper) vs. 13 (6, 20)), their median relative intensity (1.27 (1.02, 1.65) vs. 1.00 (0.77, 1.27)), the sum of the intensity of all detected peaks (1.69✕10^6^ mV (0.97✕10^6^ mV, 2.39✕10^6^ mV) vs. 0.90✕10^6^ mV (0.27✕10^6^ mV, 1.62✕10^6^ mV)) and a decrease in the measurement error (249 ppm (186 ppm, 338 ppm) vs. (289 ppm (213 ppm, 388 ppm)) (all p-values < 0.0001) when compared to incorrectly identified spectra (**Figure 1** and **Supplementary Figures S1** and **S2**). In order to account for reproducibility, we included the fraction of reproducibly detected peaks between technical replicates as fifth quality measure. These five features were henceforth used to evaluate the spectral quality in the dataset.

When comparing correctly to incorrectly identified spectra we observed, over all phylogenetic groups, a small increase in total number of peaks (173 (146, 203) vs. 163 (128, 206), p-value < 0.0001)). However, when comparing within each phylogenetic group, and especially for spectra acquired on the microflex Biotyper, we did not observe a beneficial effect of an increased total number of peaks (**Supplementary Figure S1**). Therefore, we did not include the number of peaks as a quality measure. The fraction of peaks > 10’000 Da (30.5% (23.0%, 38.2%) vs. 31.6% (18.1%, 42.4%), p-value = 0.91) and the m/z value at the 90^th^ percentile (15,323 Da (13,304 Da, 16,128 Da) vs. 15,387 Da (11,394 Da, 16,264 Da), p-value = 0.07) were comparable between correctly and incorrectly identified spectra, respectively.

In the following, we evaluated which sample preparation yielded highest quality spectra, over all phylogenetic groups, for unknown samples and within each phylogenetic group separately.

### Mass Spectral Quality Improvement With Different Sample Preparation Methods

In order to identify the best sample preparation, we first tested three different protocols. Over all phylogenetic groups, we found the ‘25% FA overlay’ method yielded the highest spectral quality.

We observed the median relative intensity of ribosomal marker peaks (1.49 (1.14, 1.91) vs. 1.27 (0.97, 1.73), p-value = 0.025), the sum of the intensity of all detected peaks (2.16✕10^6^ mV (1.53✕10^6^ mV, 2.73✕10^6^ mV) vs. 1.80✕10^6^ mV (1.22✕10^6^ mV, 2.36✕10^6^ mV)) and the fraction of reproducibly detected peaks (74.0% (66.0%, 80.1%) vs. 69.5%, (59.7%, 77.7%)) to be higher for spectra acquired under the ‘25% FA overlay’ method compared to the ‘smear’ method (p-values < 0.0001). Furthermore, we observed less variation when comparing the number of ribosomal marker peaks detected (22 (19, 25) vs. 22 (16, 25)) for spectra acquired under the ‘25% FA overlay’ method compared to the ‘smear’ method.

Spectra acquired with the ‘simple protein extraction’ method yielded overall lower values for these measures (‘median relative intensity of the ribosomal marker peaks detected’: 1.17 (1.03, 1.37); ‘sum of the intensity of all detected peaks’: 1.22✕10^6^ mV, (0.74✕10^6^ mV, 2.14✕10^6^ mV); ‘number of ribosomal marker peaks detected’: 19 (12, 23)) when compared to spectra acquired under the ‘smear’ method (p-values < 0.0001), except for the fraction of reproducibly detected peaks, where we observed higher values for spectra acquired under the ‘simple protein extraction’ (73.7%, (65.3%, 82.2)) when compared to spectra acquired under the ‘smear’ method (p-value < 0.0001). The accuracy of identification by PAPMID™ generally follows quality measures, with the highest fraction of correctly identified spectra under the ‘25% FA overlay method’ (**Figures 1**, **Supplementary Figure S3**).

### Increased Bacterial Age Decreased Spectral Quality

**Figure 2:**
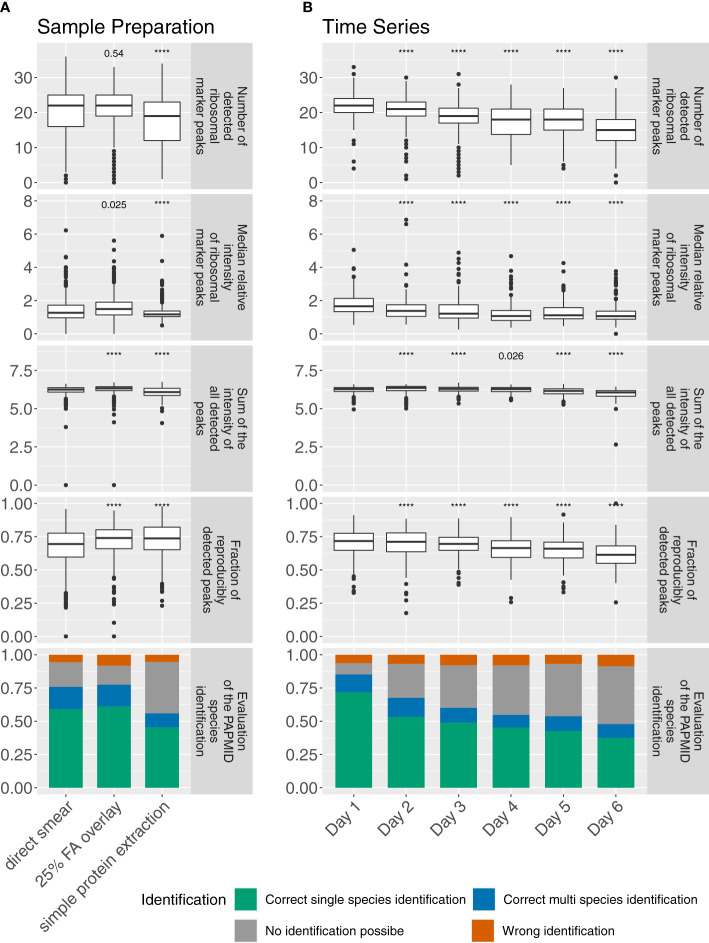
**(A)** Comparison of different sample preparation protocols across all 47 bacterial isolates, (including 3 biological replicates and 4 technical replicates for each strain and protocol) and **(B)** age of the bacterial colony (47 bacterial isolates, 4 technical replicates for each strain and day) for spectra acquired on a microflex Biotyper MALDI-TOF MS system. ‘****’p-value < 0.0001.

In order to assess how the age of a bacterial colony influences mass spectral quality, we measured the strains in our dataset after varying incubation time. We found a younger bacterial colony to be associated with a higher mass spectral quality. Increasing colony age had a negative impact on spectral quality with less ribosomal marker peaks detected (19.5 (17, 22) vs. 22 (20, 24)) and with a lower relative intensity (1.24 (0.96, 1.75) vs. 1.65 (1.34, 2.12)), and a lower fraction of reproducibly detected peaks (69.5% (64.8%, 74.6%) vs. 71.2% (64.6%, 77.6%)) after three days when compared one day incubation time (p-values < 0.0001) (**Figure 2**).

The accuracy of identification by PAPMID™ generally follows quality measures, with an increasing number of spectra not being identified, and decreasing spectral quality over the time period.

### The Amount of Bacterial Colony Material Applied Has a Significant Impact on Spectral Quality

In order to identify the best preparation procedure, we tested varying concentrations of the bacterial sample applied to the steel target plate.

**Figure 3:**
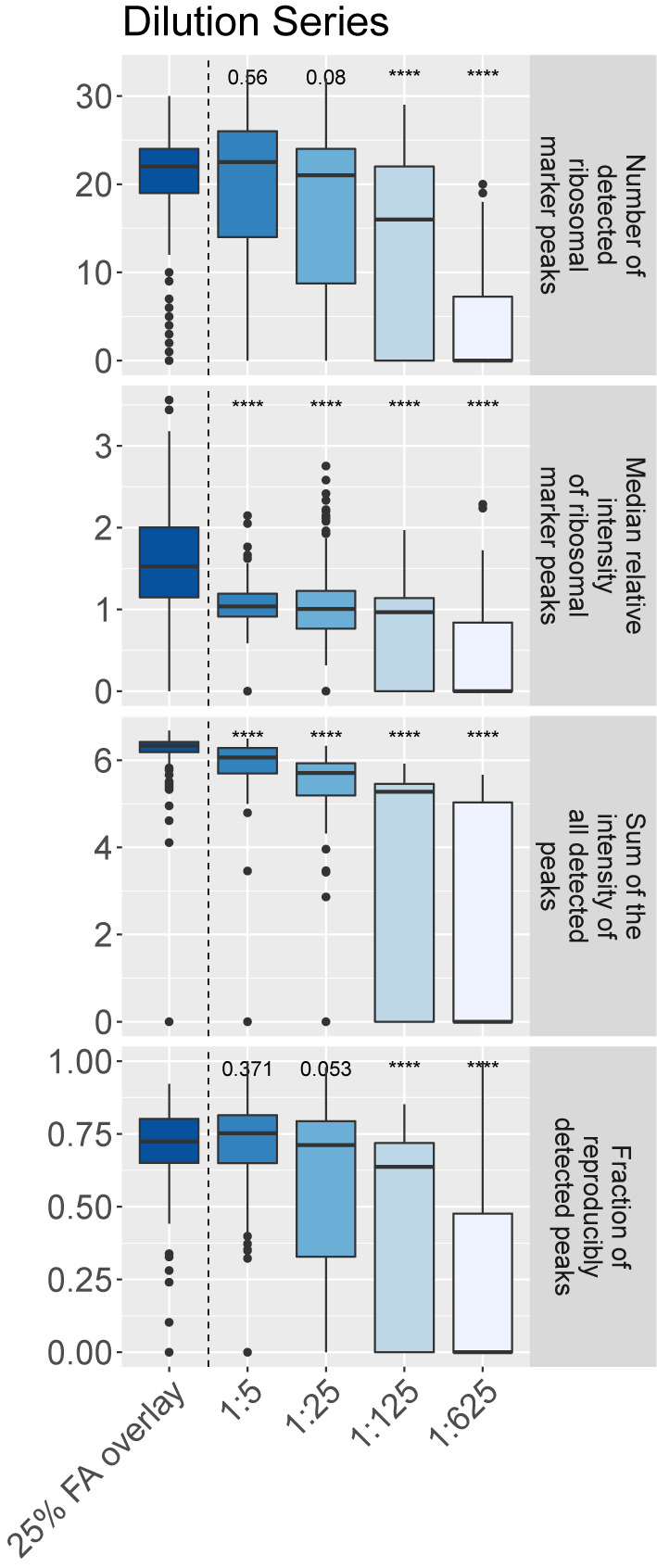
Comparison of the amount of bacterial colony material applied onto a steel target plate for spectral quality (16 bacterial isolates, 3 biological and 4 technical replicates per dilution and strain). Spectra were acquired on a microflex Biotyper MALDI-TOF MS system. ‘****’p-value < 0.0001.

Over all phylogenetic groups, we found that diluting the bacterial sample 1:5 did not decrease the number of ribosomal marker peaks detected (22.5 (14, 26) vs. 22 (19, 24)), nor the fraction of reproducibly detected peaks (75.2% (65.0%, 81.4%) vs. 72.4% (65.0%, 80.1%)) when compared to spectra acquired under the ‘25% FA overlay’ method (**Figure 3**).

However, we observed a decreased median intensity of the ribosomal marker peaks (1.06, (0.94, 1.21) vs. 1.53 (1.16, 2.02)) and a decreased sum of the intensity of all detected peaks (1.17✕10^6^ mV (0.50✕10^6^ mV, 1.92✕10^6^ mV) vs. 2.15✕10^6^ mV (1.53✕10^6^ mV, 2.64✕10^6^ mV)) for 1:5 diluted samples when compared to samples processed using the ‘25% FA overlay’ method (p-values < 0.0001).

Diluting bacterial colony material 1:25 or more generally decreased mass spectral quality (‘number of ribosomal marker peaks detected’: 21 (8.75, 24); ‘median intensity of the ribosomal marker peaks: 1.07 (0.95, 1.29); ‘sum of the intensity of all detected peaks’: 0.51✕10^6^ mV, (0.16✕10^6^ mV, 0.85✕10^6^ mV)). However, we found taxon specific effects e.g. with *Burkholderia* yielding highest quality spectra with the highest number of ribosomal marker peaks detected with an additional dilution to 1:25 (**Supplementary Figures S5** and **S6**).

### Calibration Is Crucial

**Figure 4:**
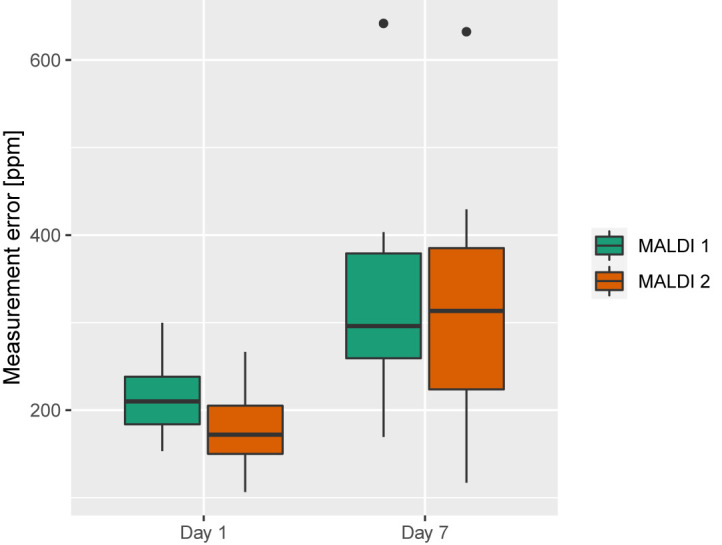
Measurement error for Day 1 and Day 7 after calibration (1 bacterial isolate, 3 biological and 4 technical replicates per day).

All MALDI-TOF MS were externally calibrated in a routine setting and the effect of calibration has been previously investigated (Mitchell et al., 2015). Here, we tested the impact of time between calibration and the measurement on measurement precision. We found that the measurement error increased with time after calibration, (Day 1: 194 ppm (166 ppm, 235 ppm) vs. Day 7: 296 ppm (236 ppm, 379 ppm)) (p-value < 0.0001) (**Figure 4**).

### Major Differences in Spectra Quality Between Bacterial Taxa

**Figure 5:**
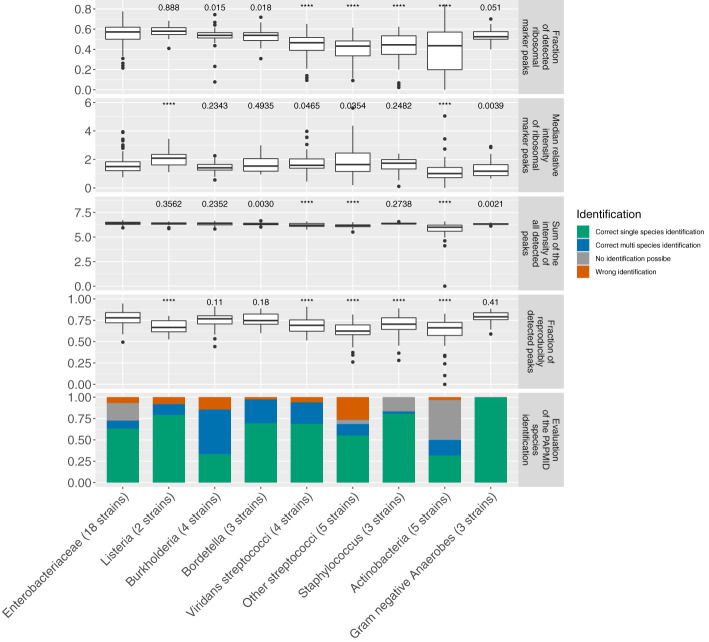
MALDI-TOF mass spectra features and species identification of spectra acquired with the ‘25% FA overlay’ method and after one day of incubation on a microflex Biotyper (3 biological and 4 technical replicates per strain). ‘****’p-value < 0.0001.

Testing whether the mass spectral quality is sufficient for spectra acquired with the ‘25% FA overlay’ method for all bacterial taxa, we found important differences (**Figure 5**, **Supplementary Figure S7**). *Enterobacteriaceae* was the biggest family in our dataset (18 strains) and strains within this family generally yielded rich MALDI-TOF mass spectra with a high fraction of ribosomal marker peaks detected 57.1%, (50.0%, 61.9%)). For statistical analyses, we used *Enterobacteriaceae* as a reference group (**Figure 5**, **Supplementary Figure S7**). On both MALDI-TOF MS systems, we found Gram positive bacteria generally yielded lower quality spectra than the Gram negative strains, with a lower fraction of ribosomal marker peaks detected (46.1% (34.7%, 53.6%) vs. 55.0% (50.0%, 61.9%), a lower sum of the intensity of all detected peaks (1.64✕10^6^ mV (1.11✕10^6^ mV, 2.39✕10^6^ mV) vs. 2.38✕10^6^ (1.91✕10^6^ mV, 3.05✕10^6^ mV)) and a lower fraction of reproducibly detected peaks (66.7% (60.2%, 73.9%) vs. 77.7% (72.0%, 82.8%)) (p-values < 0.0001). *Actinobacteria* and streptococci other than viridans streptococci yielded lowest quality MALDI-TOF mass spectra with the lowest fraction of ribosomal marker peaks detected (43.6% (19.8% - 57.1%), 43.2% (33.5% - 48.3%), respectively) and the lowest fraction of reproducibly detected peaks (66.1% (57.2% - 72.4%), 62.3% (58.2% - 69.5%), respectively).

Among the generally lower performing Gram positive bacteria and against the general trend, we detected the highest median fraction of detected ribosomal marker peaks for *Listeria* (58.0% (54.5% - 61.4%)), whereas Gram negative anaerobes yielded the highest fraction of reproducibly detected peaks (79.0%, (75.6% - 83.7%)).

### Differences Between MALDI-TOF MS Databases

In order to evaluate different available databases we compared spectra acquired on the microflex Biotyper to the MALDI Biotyper database (MALDI Biotyper Compass Library, Revision E (Vers. 8.0, 7854 MSP, RUO)) and spectra acquired on the Axima Confidence system to the VitekMS database (v3.2) for species identification. All spectra compared were acquired under the ‘25% FA overlay’ method. Please note that, while spectra were compared to the entire latter two databases for species identification, they were compared only to a subset of entries or subtyping modules of the PAPMID™ database.

**Figure 6:**
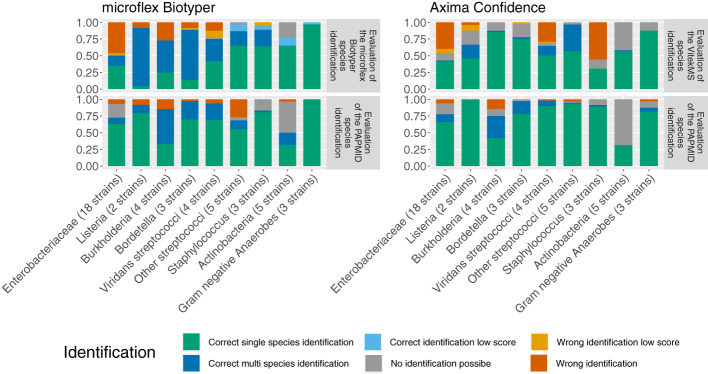
Evaluation of bacterial species identification. MALDI-TOF mass spectra were acquired on the microflex Biotyper (left) and the Axima Confidence (right) with the ‘25% FA overlay’ method and after one day of incubation (3 biological and 4 technical replicate for each strain). Species identification was performed by the three different databases MALDI Biotyper (upper left), the VitekMS (upper right) and PAPMID™ (lower panels). Color Code: green: correct single species identification: dark blue: correct identification, multiple species above threshold or VitekMS Identification Type ‘Low Discriminatory’; light blue: correct identification, MALDI Biotyper log score < 2, grey: no identification possible, yellow: wrong species identified, low security (MALDI Biotyper log score < 2 or VitekMS Identification Type ‘Low Discriminatory’), red: wrong species identified, high security (MALDI Biotyper log score > 2, VitekMS Identification Type ‘Single Choice’ and single species identification using a marker based approach).

Neither the MALDI Biotyper nor the VitekMS database cover all species included in this study (**Supplementary Table S1**). Spectra of strains belonging to species missing in these databases are often wrongly identified as closely related species represented in the database (**Figure 6**). The MALDI Biotyper database covers more species represented in our strain collection than the VitekMS DB (**Supplementary Table S1**) and more often results in a correct species assignment (**Figure 6**). However, comparison of spectra to MALDI Biotyper databases can lead to ambiguous results with multiple species yielding Scores > 2.0.

We observed the biggest difference between the MALDI Biotyper and the VitekMS database for *Staphylococcus* spectra, including spectra of the species *S. aureus*, *S. argenteus* and *S. schweitzeri*, with correctly identified species in 94.4% of spectra using the MALDI Biotyper database compared to 30.6% using the VitekMS database.

Species identification by the PAPMID™ yielded more often correct single species identification for spectra acquired on the Axima Confidence device than for spectra acquired on the microflex Biotyper device.

### Increased Mass Spectral Quality Increased Species Identification Accuracy

In order to understand the impact of mass spectral quality on species identification accuracy in more detail we looked at three species complexes, namely the *Burkholderia cepacia complex*, viridans streptococci and the *Enterobacter cloacae complex*, and exclusively considering species which are covered by all three databases examined in this study. We split the spectra of these three complexes into three equal groups according to the sum of the intensity of all detected peaks in each spectrum, using this as a universally applicable proxy for spectral quality. We observed that with increasing intensity, the number of detected ribosomal marker peaks, their median relative intensity, the reproducibility and measurement precision increase, suggesting that these quality features are correlated.

**Figure 7:**
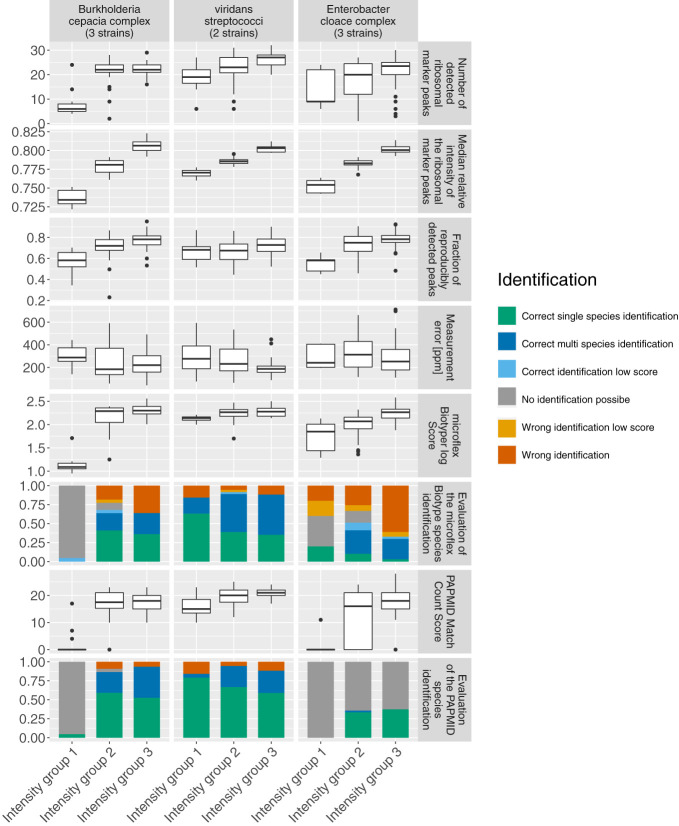
Spectra quality features and evaluation of species identifications grouped by the sum of the intensity of all detected peaks. Spectra acquired on the microflex LT/LH ‘smart’. Color Code: green: correct single species identification; dark blue: correct identification, multiple species above threshold; light blue: correct identification, MALDI Biotyper log score < 2; grey: no identification possible, yellow: wrong species identified, low security (MALDI Biotyper log score < 2), red: wrong species identified, high security (MALDI Biotyper log score > 2 and single species identification using a marker based approach).

Importantly, we found a larger fraction of correctly identified species with a higher confidence level (MALDI Biotyper log score) with increasing spectral quality (**Figure 7** and **Supplementary Figure S19**). As an exception, we observed within the *Enterobacter cloacae complex*, an increase of incorrectly identified spectra using the MALDI Biotyper database with an increasing spectral quality.

### Taxon-Specific Sample Preparation for Highest Spectral Quality

Following the inherent differences in mass spectral quality between the phylogenetic groups (**Figure 5**) we hypothesise taxon-specific improvement of spectral quality when using different sample preparation, quantity, and age of the bacterial colony. In order to assess these we have compared the sample preparation conditions evaluated in this study, for each group separately (**Supplementary Figures S5, S6, S8–S16**). Here, we suggest optimized taxon-specific sample preparation and handling protocols in order to achieve optimal spectral quality. We summarised the optimal sample preparation and bacterial colony age per group which yielded good quality spectra (**Table 2**).

**Table 2:** Optimal sample protocol and bacterial age summarised per phylogenetic group.

Group	Protocol	Day
***Enterobacteriaceae***	25% FA overlay	Day 1
***Listeria***	25% FA overlay/Dilute 1:5	Day 1/2/3/4
***Burkholderia***	Dilute 1:25	Day 2
***Bordetella***	25% FA overlay/Dilute 1:5	Day 1/2
***Streptococcus***	Simple protein extraction	Day 1/2/3/4/5
***Staphylococcus***	Dilute 1:5/Simple protein extraction	Day 1/2
***Actinobacteria***	Simple protein extraction	Day 1
**Gram - Anaerobes**	Dilute 1:5	Day 1/2

## Discussion

In this study we determined MALDI-TOF mass spectra quality features, associated with correct identification and showed that these features can be increased in routine diagnostics by adapting sample preparations protocols.

Comparing the spectra quality yielded by varying sample preparations we found that over all phylogenetic groups and for unknown samples, measuring bacterial samples at a young age and overlaying the sample with 25% formic acid yielded the best quality spectra. As *Enterobacteriaceae* was the biggest group in our dataset, it had the strongest influence on the optimal sample preparation protocols when we analysed all strains congruently. Nonetheless, also when analyzing the impact of different sample preparation protocols for each group separately, the ‘25% FA overlay’ method was amongst the best performing methods for most phylogenetic groups and with little hands-on time.

Over all phylogenetic groups, we observe the highest mass spectral quality after one overnight culture, followed by a decrease in mass spectral quality with increasing bacterial colony age. However, slower growing bacteria might require a longer incubation time before sufficient bacterial material can be transferred onto a target plate and before entering a phase of exponential growth, where ribosomal proteins are highly abundant (Fenselau and Demirev, 2001).

We find that, over all phylogenetic groups, diluting the bacterial sample 1:5 does not decrease mass spectral quality and a dilution step can in fact increase the spectral quality for certain taxa.

Overlaying the bacterial colony material with 25% formic acid does not increase spectral quality for all phylogenetic groups and can in fact often be omitted, and these samples can be prepared using the ‘direct smear’ method when the taxon of an isolate is known. On the other hand, not all phylogenetic groups yielded good-quality spectra even when overlaying the sample with 25% formic acid, most notably *Actinobacteria*. Here, a ‘simple protein extraction’ might be required to detect intracellular proteins (Alatoom et al., 2011).

Summarizing our sample preparation experiments, we encourage laboratories working in routine diagnostics to measure unknown microorganisms after one night of growth, with little bacterial colony material, and overlaying each spot with 25% formic acid. If the spectra acquired using this protocol do not yield satisfying identification results, we furthermore propose the application of taxon-specific protocols. These can also be used to obtain optimal quality mass spectra for subtyping.

To define mass spectral quality, we analysed several spectral features among which we identified the following five as best proxies: (i) number of ribosomal marker peaks detected, (ii) median relative intensity of ribosomal marker peaks, (iii) sum of the intensity of all detected peaks, (iv) measurement precision, and (v) reproducibility of all peaks. The first four were increased in spectra which were correctly identified with all three databases when compared to incorrectly identified spectra. The effect of these features is more pronounced when spectra are acquired on the Axima confidence than on the microflex Biotyper. Incorrectly identified Axima Confidence spectra appear to be signal poor with a low total number of peaks (**Supplementary Figure S2**). Incorrectly identified microflex Biotyper spectra can harbour a high number of peaks, but are sparse in ribosomal marker masses and sum of the intensity of all detected peaks, which suggests that these spectra are noisy (**Supplementary Figure S1**). This is also reflected in the higher number of false positive hits in ribosomal marker masses leading to a higher fraction of wrongly identified microflex Biotyper spectra than Axima Confidence spectra when compared to the PAPMID™ database. As hardware settings, such as the tension of the detector, might affect the total number of peaks, it remains unclear whether the observed trends hold true for all microflex Biotyper devices. A study involving multiple devices is required to assess this question.

When using the ‘25% FA overlay’ method, we found a median of 74.0% of peaks reproducibly detected in technical replicates of the same sample (**Figure 2**). This measure assesses the reproducibility of picked peaks with which we decided to work with, as they are the bases for species identification. This measure of reproducibility is different from the pearson correlation, comparing the shapes of two or more spectra (Zhang et al., 2014; Oberle et al., 2016). A reproducible detection of 75% of the picked peaks in a spectrum with 100 peaks, would mean that 75 peaks were detected in at least 3 out of 4 technical replicates of the same measurement. By using optimal sample preparation methods, we can increase the number of reproducibly detected peaks. These reproducibly detectable peaks could potentially be used as marker peaks, additional to ribosomal subunit masses and for spectra identification, further increasing the resolution of this method.

We observed the measurement error to increase with increasing time after calibration and therefore advise for frequent calibration of MALDI-TOF MS devices.

Microbiology taxonomy is in flux and many bacterial species have been newly described or have changed the genus in recent years (Janda, 2020). It is hardly possible for any diagnostic database to be up to date at every moment in time. We would like to emphasise that we have included strains in this study which pose difficulties for bacterial species identification and that bacterial species identification by MALDI-TOF MS is highly accurate in routine diagnostics (Croxatto et al., 2012). The challenges posed by the species included in this study are known and also clearly communicated by the MALDI-TOF MS manufacturers by e.g. displaying a warning message indicating which species cannot, or not reliably be distinguished from one another.

As the MALDI Biotyper database covers more of the species included in this study than the VitekMS database, spectral assignment from this database more often results in a correct species identification (**Figure 6**). This is most remarkable for spectra of the *S. aureus* complex, where the MALDI Biotyper database includes all three species (*S. aureus*, *S. argenteus* and *S. schweitzeri*), whereas the VitekMS database lists only *S. aureus* (**Supplementary Table S1**). However, interpreting the MALDI Biotyper species identification is not always trivial as multiple species can yield a log score > 2, which is used as a threshold for the assignment ‘highly confidence identification’.

Importantly, we have shown that an increased spectra quality can increase the accuracy of species identifications by all three databases. However, against the general trend, the number of incorrectly identified spectra increases with increasing spectra quality for species of the *Enterobacter cloacae complex* analysed with the MALDI Biotyper database. A possible explanation could be the MALDI Biotyper database frequently assigning the more frequent sister species *E. cloacae sensu stricto*.

We find *Actinobacteria* yielding the lowest spectra quality of all phylogenetic groups analysed in this study. When comparing spectra of this group to the PAPMID™ database we find less often correctly identified spectra, compared to the other phylogenetic groups. For *Actinobacteria* only few ribosomal marker peaks can be detected, which makes distinction solely based on these, difficult. For this group, species identification using a pattern matching approach, applied by the MALDI Biotyper and the VitekMS database, more often yielded correct results. As it remains unclear which proteins form the basis of this species identification and how these vary between closely related species, it is possible that discrimination between closely related species might be challenging within *Actinobacteria* using a pattern matching approach.

MALDI-TOF mass spectra quality might be influenced by factors not considered in this study including: (i) hardware factors such as the age and intensity of the laser; (ii) the type of MALDI-TOF MS target plates and matrix used; (iii) culturing variables such as the agar media used or the atmosphere in which bacterial isolates are grown; (iv) spectra acquisition settings such as the number of laser shots applied and spectra averaged per measurement and (v) factors considering technical knowledge on acquiring MALDI-TOF mass spectra including regular training of staff and quality control of MALDI-TOF MS measurements. In order to assess and standardise MALDI-TOF mass spectral quality in routine diagnostics, a broader study comparing spectra acquired in multiple laboratories by different personnel is required.

The reliable detection of marker peaks in clinical routine would allow for higher resolution typing based on MALDI-TOF mass spectra, also distinguishing between closely related species e.g. within the *Klebsiella pneumoniae complex*, the *Staphylococcus aureus complex* and within viridans streptococci. An effective standardisation in culture conditions and spectra quality assessment might help the automation process of colony picking and mass spectral acquisition. Using a marker based approach for identification, we can congruently query spectra acquired on different MALDI-TOF MS systems around the world. Using the potential of routinely generated MALDI-TOF MS data for sublineage detection would open up new avenues of disease control by tracing the spread of important sub-lineages in real time with little additional effort.

## Data Availability Statement

The datasets presented in this study can be found in online repositories. The names of the repository/repositories and accession number(s) can be found in the article/**Supplementary Material**.

## Author Contributions

AC, AE, and VP designed and planned the study. AC conducted the experiments. AC, FF, and VP performed bioinformatic analysis. AC, AE, FF, and VP critically reviewed the manuscript. All authors contributed to the article and approved the submitted version.

## Funding

This study was supported by a “Personalized Health” at ETHZ (D-BSSE) and University of Basel grant (PMB-03-17) and a Doc.Mobility Fellowship by the Swiss National Science Foundation (P1BSP3-184342).

## Conflict of Interest

VP and FF are employed by Mabritec AG.

The remaining authors declare that the research was conducted in the absence of any commercial or financial relationships that could be construed as a potential conflict of interest.
