# Supplementary material for: Factors Associated With MALDI-TOF Mass Spectral Quality of Species Identification in Clinical Routine Diagnostics
Source: Front Cell Infect Microbiol. 2021 Mar 16;11:646648. doi: 10.3389/fcimb.2021.646648 (PMC8007975; doi:10.3389/fcimb.2021.646648)
Supplement: Supplementary file 1 [file DataSheet_1.pdf]

Supplement

Supplementary Methods 1: Short description of the spectra features queried for each spectrum.

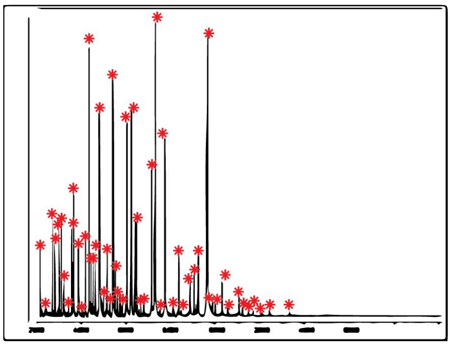

Total Number of peaks: The total number of peaks detected by the microflex Biotyper / VitekMS Software using default settings.

The accuracy and resolution of MALDI-TOF MS increases the more informative peaks can be detected. However, too noisy spectra cannot be interpreted.

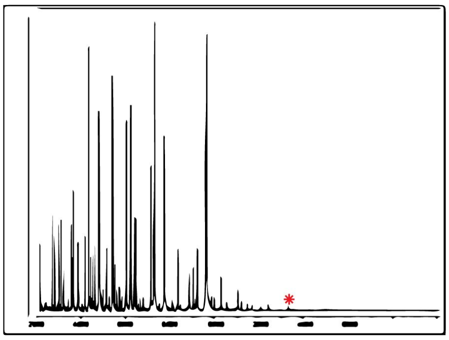

Peak with the highest m/z value: The highest m/z value to which a peak was assigned to by the microflex Biotyper / VitekMS Software using default settings.

We included the 'highest peak detected' to measure the mass range covered by the spectrum. However, the highest peak detected often corresponds to an artifact. We therefore focus on the peak at the 90th percentile.

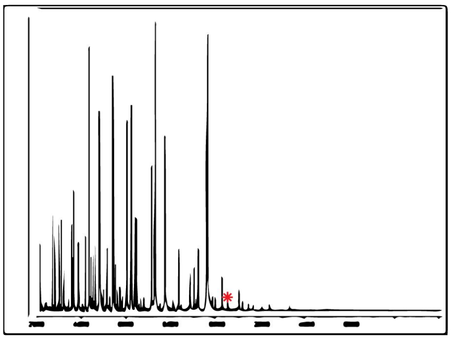

Peak at the 90th percentile of m/z values: The m/z value at the 90th percentile considering all peaks assigned by the microflex Biotyper / VitekMS Software using default settings.

In order to measure the mass range covered by the spectrum and omitting technical artefacts we included the peak at the 90th percentile.

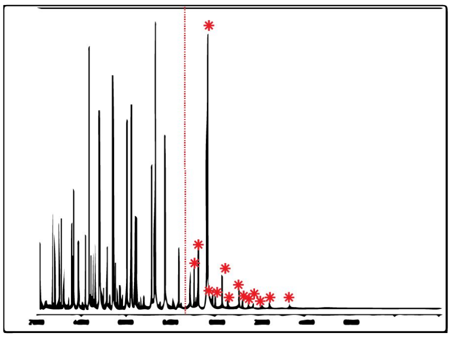

Fraction of high peaks: (Number of peaks with a m/z value > 10'000) / **(**Total number of peaks)

We included this measure as an estimate how balanced a MALDI-TOF mass spectrum is.

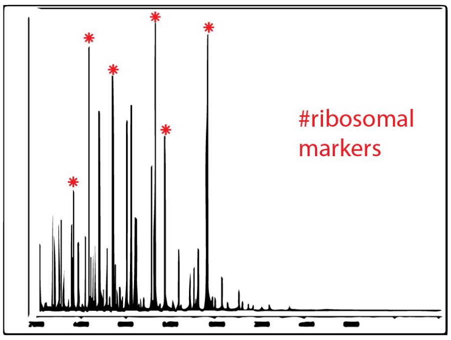

Number of ribosomal marker peaks detected: The number of predicted ribosomal subunits, which could be detected in a MALDI-TOF mass spectrum within an error of 800ppm. Each spectrum was queried for the m/z values calculated from the genomic sequence of the same strain.

The accuracy and resolution increase with increasing number of phylogenetic marker peaks, which can reproducibly be detected.

As not all bacterial strains encode the same number of ribosomal marker in the mass range of 2’000 - 20’000 Da, we normalised this value by dividing the number of detected phylogenetic markers through the number of predicted phylogenetic markers in the MALDI-TOF MS mass range when comparing values between the phylogenetic groups (**Fraction of phylogenetic markers detected**).

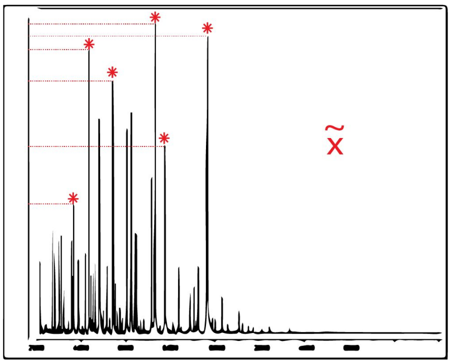

Median relative intensity of the detected ribosomal marker peaks: The median, relative intensity of the detected marker peaks. In order to make intensity values between spectra comparable, all intensities were normalised by dividing through the median intensity within a spectrum.

The median, relative intensity of ribosomal subunits was included as a measure for trustworthiness of these markers. As ribosomal subunits occur in high copy numbers in the replicating bacterial cells measured, these peaks should be high, relative to other protein peaks. Low relative intensities might be evidence for false positive hits.

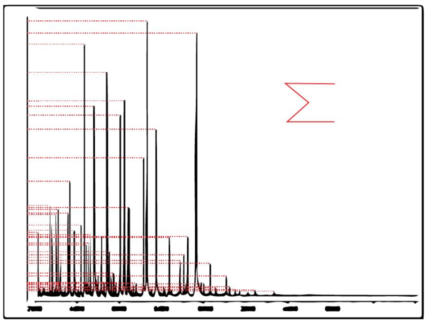

Sum of the intensity of all detected peaks: Sum of the (not normalised) intensity values of all peaks assigned by the microflex Biotyper / VitekMS Software using default settings.

We included the total intensity of all peaks as a measure for how much signal was detected. This is not the same value as the ‘Total Ion Current’ as this would also include baseline signals or signals under the signal / noise threshold applied whereas here we focus on the total intensity of the peaks exclusively. However, these two measures will strongly correlate.

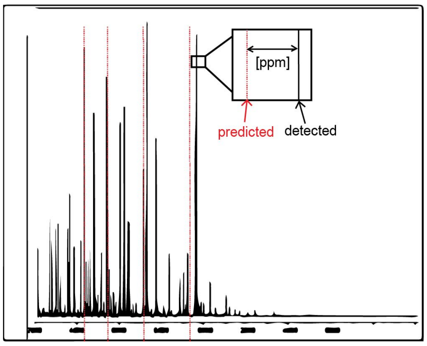

Measurement error: The mean distance between the predicted m/z value of a ribosomal marker peak and the detected peak. As the measurement precision in MALDI-TOF mass spectra is depending on the mass range, these values are given in ppm (parts per million) and absolute values (Daltons). Marker masses were queried for each spectrum, considering an error range of 800ppm.

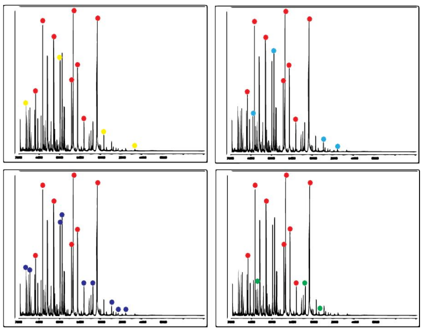

Fraction of reproducibly detected peaks: Number of peaks which were detected in more than half of all technical replicates / Total number of peaks per spectrum.

With an increasing number of reproducible detected phylogenetic marker masses, the resolution of MALDI-TOF MS can further increase.

Table S1: Summarising, which species are included in which MALDI-TOF MS databases

**Species**	**microflex Biotyper** (MALDI Biotyper Compass Library, Revision E (Vers. 8.0, 7854 MSP, RUO))	**VitekMS DB** (v3.2)	**marker based DB**
Klebsiella pneumoniae	Yes	Yes	Yes
Klebsiella oxytoca	Yes	Yes	Yes
Klebsiella grimontii	No	No	Yes
Klebsiella michiganensis	No	No	Yes
Klebsiella aerogenes	Yes	Yes (as *Enterobacter aerogenes*)	Yes
Klebsiella variicola	Yes	Yes	Yes
Klebsiella quasivariicola	No	No	Yes
Klebsiella quasipneumoniae	No	No	Yes
Klebsiella huaxensis	No	No	Yes
Klebsiella africanensis	No	No	Yes
Listeria monocytogenes	Yes	Yes	Yes
Listeria ivanovii	Yes	Yes	Yes
Listeria innocua	Yes	Yes	Yes

Escherichia coli	Yes	Yes	Yes
Shigella flexneri	No	Yes (displayed as *E. coli*)	Yes
Shigella sonnei	No	Yes (displayed as *E. coli*)	Yes
Burkholderia cepacia	Yes	Yes	Yes
Burkholderia cenocepacia	Yes	Yes	Yes
Burkholderia contaminans	No	Yes	Yes
Burkholderia multivorans	Yes	Yes	Yes
Bordetella bronchiseptica	Yes	Yes	Yes
Bordetella pertussis	Yes	Yes	Yes
Bordetella parapertussis	Yes	Yes	Yes
Streptococcus pneumoniae	Yes	Yes	Yes
Streptococcus mitis	Yes	Yes	Yes
Streptococcus oralis	Yes	Yes	Yes
Streptococcus infantis	Yes	No	Yes
Streptococcus gordonii	Yes	Yes	Yes
Streptococcus gallolyticus	Yes	Yes	Yes
Streptococcus lutetiensis	Yes	Yes (as *Streptococcus infantarius ssp coli* (*Str.lutetiensis*))	Yes
Streptococcus pseudopneumoniae	Yes	Yes	Yes
Streptococcus equinus	Yes	Yes	Yes
Streptococcus dysgalactiae	Yes	Yes	Yes
Streptococcus canis	Yes	Yes	Yes
Bacteroides fragilis	Yes	Yes	Yes
Enterobacter sichuanensis	No	No	Yes
Enterobacter hormaechei	Yes	Yes	Yes
Enterobacter asburiae	Yes	Yes	Yes
Enterobacter cloacae	Yes	Yes	Yes
Enterobacter ludwigii	Yes	Yes	Yes
Staphylococcus aureus	Yes	Yes	Yes

Staphylococcus schweitzeri	Yes	No	Yes
Staphylococcus argenteus	Yes	No	Yes
Corynebacterium amycolatum	Yes	Yes	Yes
Corynebacterium urealyticum	Yes	Yes	Yes
Gardnerella vaginalis	Yes	Yes	Yes
Winkia neuii	Yes (as *Actinomyces neuii*)	Yes (as *Actinomyces neuii*)	Yes
Actynomyces israelii	Yes	Yes	Yes
Pasteurella multocida	Yes	Yes	Yes

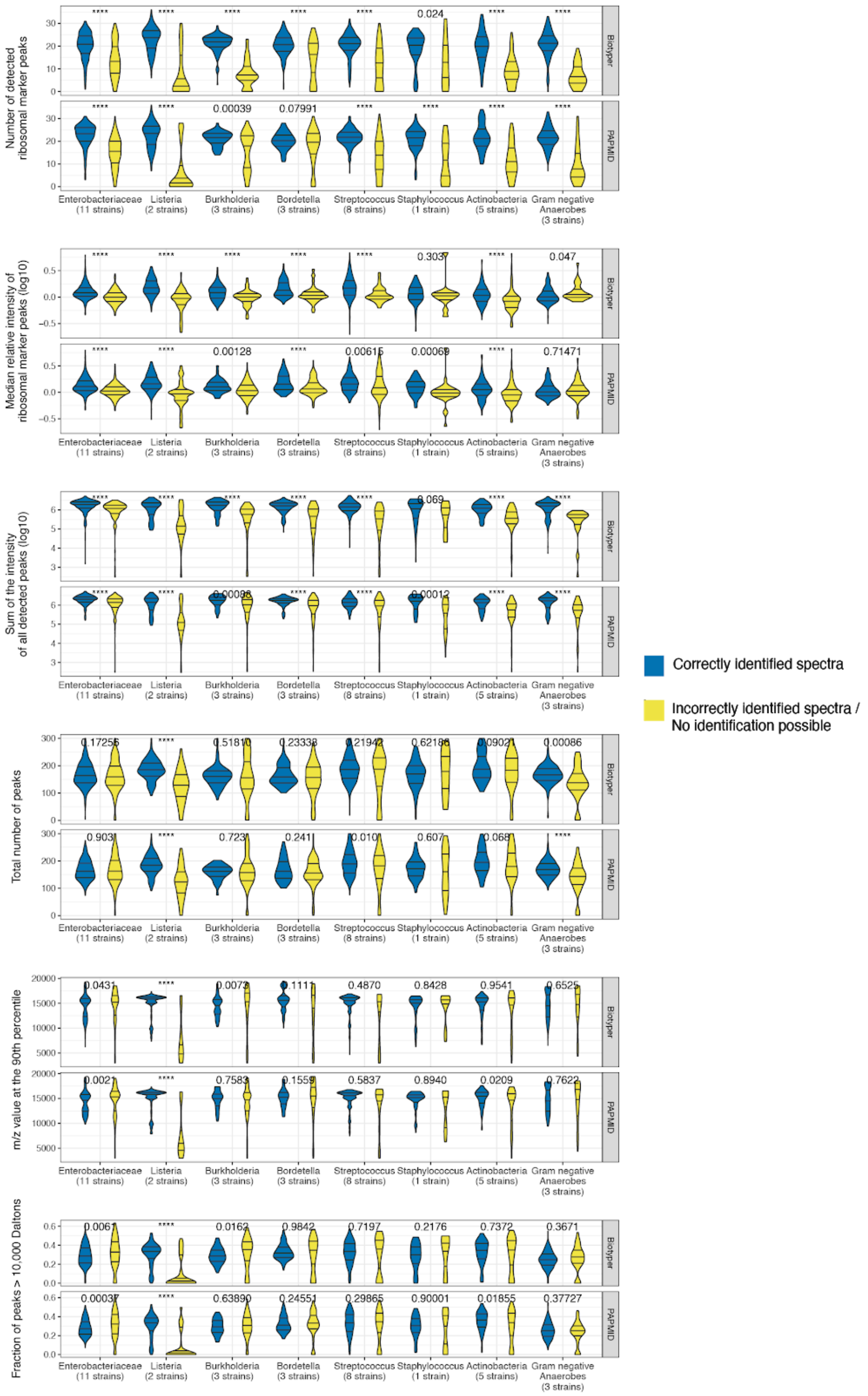

Figure S1: Spectra features (i) Number of detected ribosomal marker peaks, (ii) their median relative intensity (log10), (iii) the sum of the intensity of all detected peaks (log10), (iv) the total number of peaks, (v) the m/z value at the 90th percentile and (vi) the fraction of peaks > 10'000 Daltons compared between correctly and incorrectly identified mass spectra per phylogenetic group. Spectra were acquired on a microflex Biotyper and identified by the microflex Biotyper database (upper row) and the PAPMID™ (lower row).

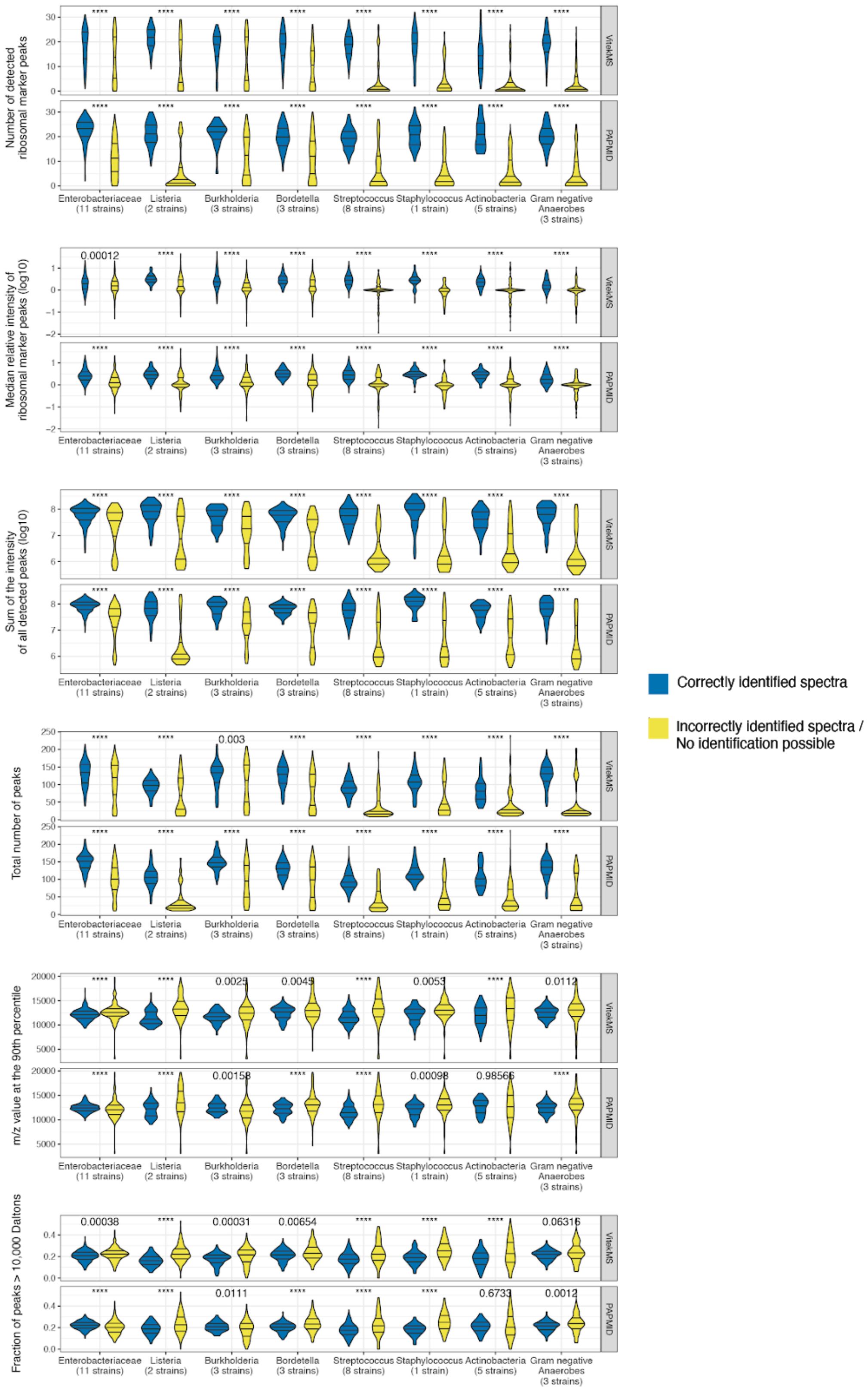

Figure S2: Spectra features (i) Number of phylogenetic marker peaks, (ii) their relative intensity, (iii) the total number of peaks, (iv) the total number of peaks, (v) the m/z value at the 90th percentile and (vi) the fraction of peaks > 10'000 Daltons compared between correctly identified spectra and spectra for which the correct species could not be identified per phylogenetic group. Spectra were acquired on a Axima Confidence and identified by the VitekMS database (upper row) and the PAPMID^{TM} (lower row).

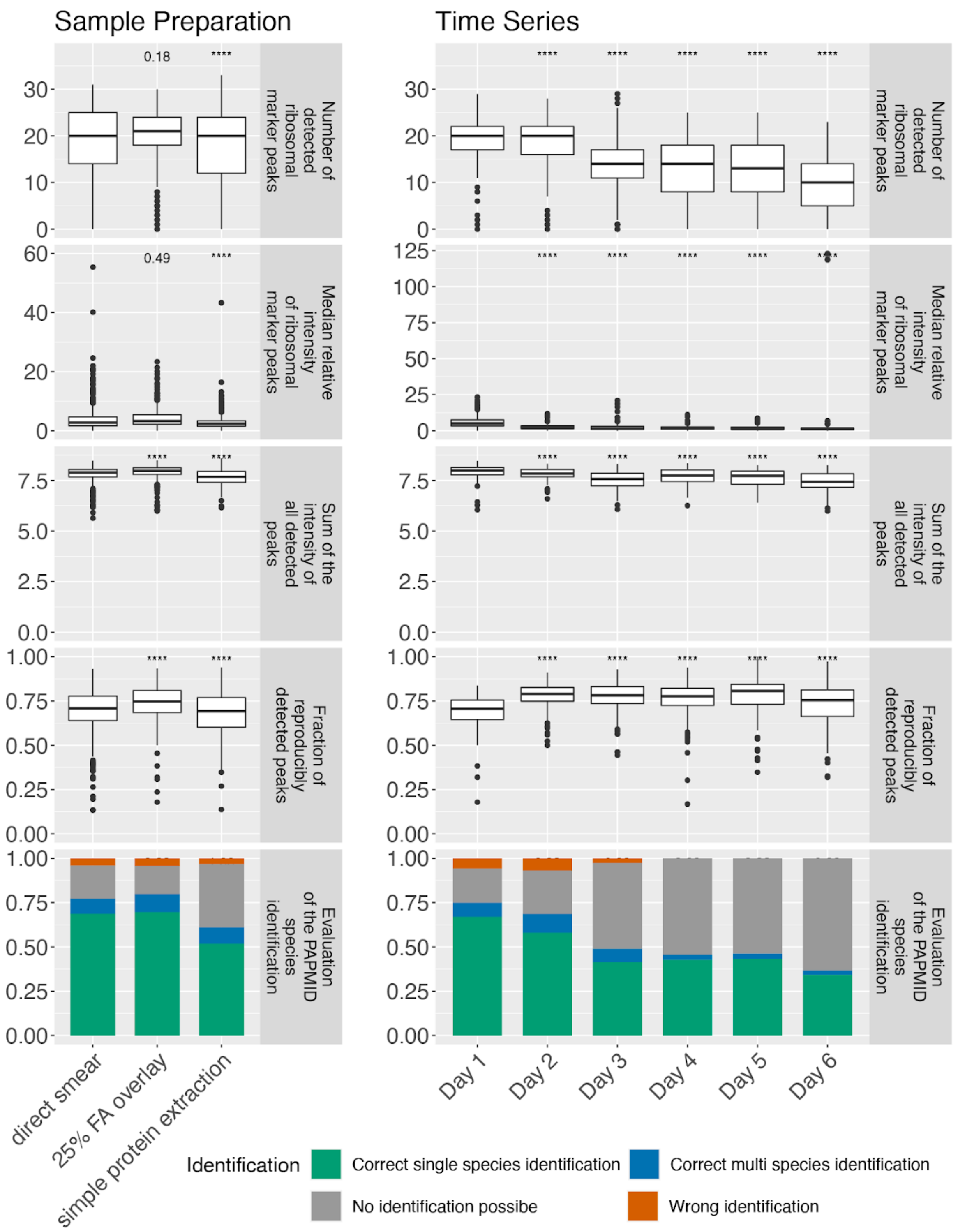

Figure S3: (A) Comparison of different sample preparation protocols across all 47 bacterial isolates, (including 3 biological replicates and 4 technical replicates for each strain and protocol) and (B) age of the bacterial colony (47 bacterial isolates, 4 technical replicates for each strain and day) for spectra acquired on an Axima Confidence MALDI-TOF MS system. '****': p-value < 0.0001.

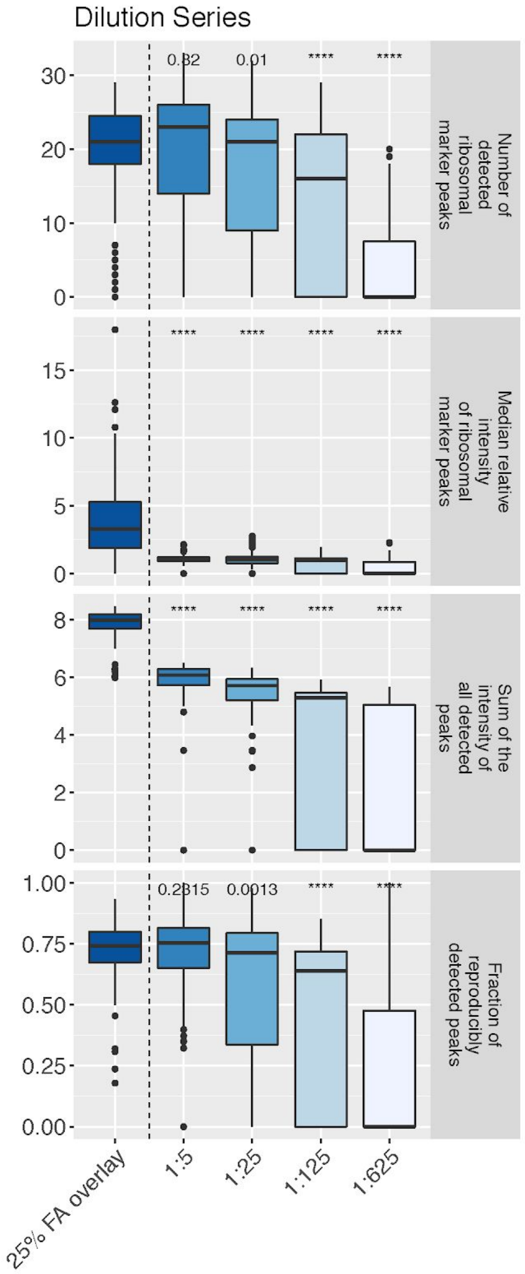

Figure S4: Comparing the amount of bacterial material applied onto a steel target plate (16 bacterial isolates, 3 biological and 4 technical replicates per dilution and strain). Spectra were acquired on a Axima Confidence MALDI-TOF MS system. '****': p-value < 0.0001.

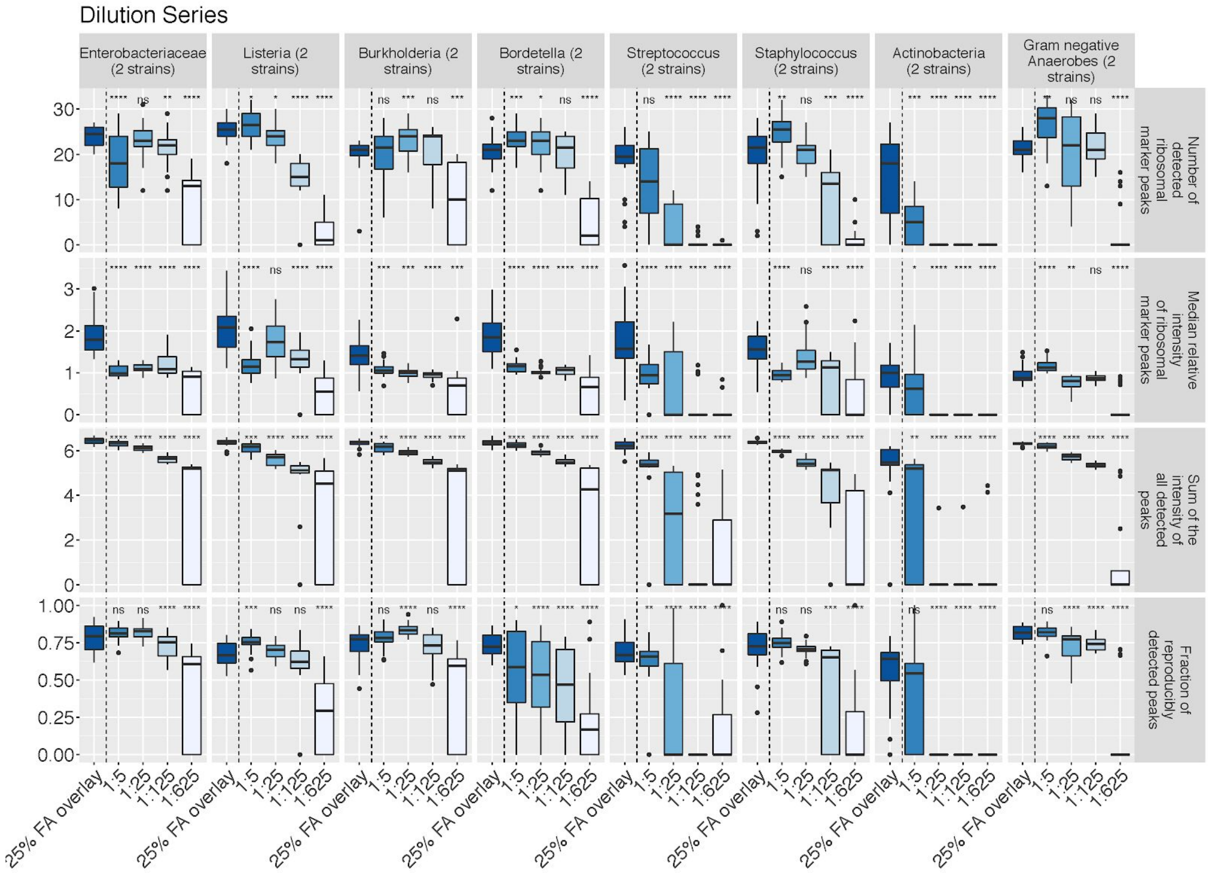

Figure S5: Comparing different dilution steps for the taxonomic groups analysed in this project (2 bacterial isolates per phylogenetic group, 3 biological and 4 technical replicates per dilution and strain). Spectra were acquired on a microflex Biotyper MALDI-TOF MS system.

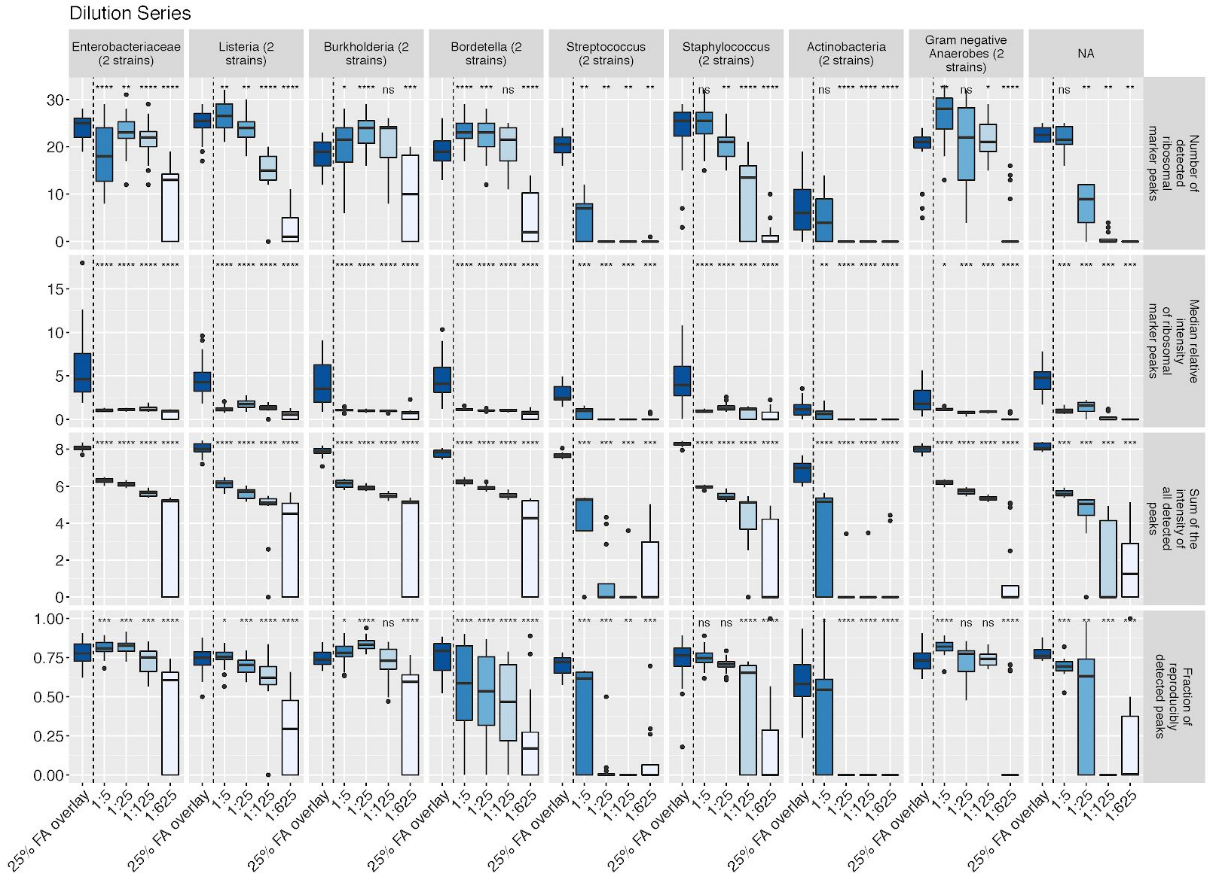

Figure S6: Comparing different dilution steps for the taxonomic groups analysed in this project (2 bacterial isolates per phylogenetic group, 3 biological and 4 technical replicates per dilution and strain). Spectra were acquired on an Axima Confidence system.

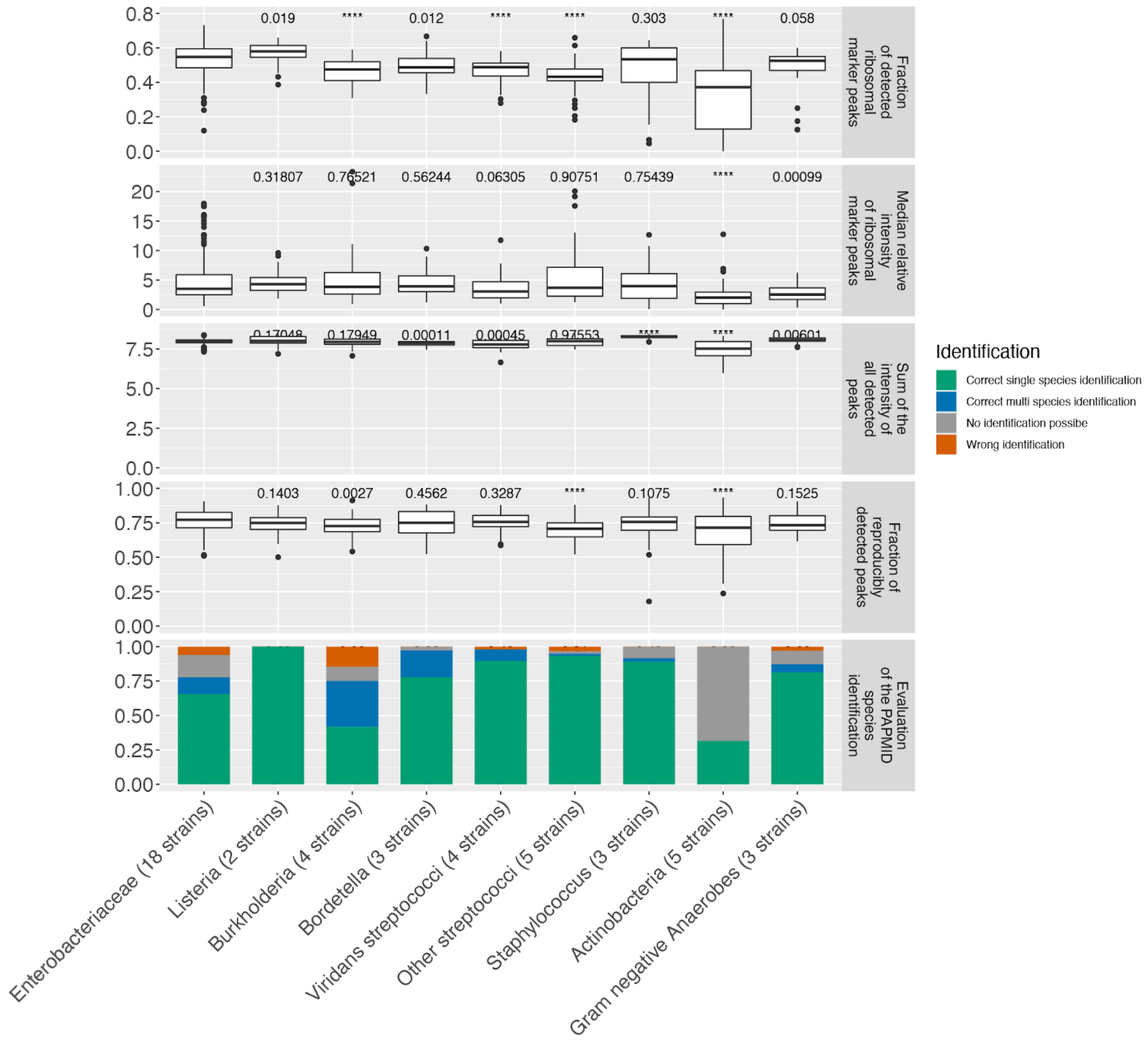

Figure S7: MALDI-TOF mass spectra features and species identification of spectra acquired with the '25% FA overlay method' and on a Axima Confidence. Spectral features depicted are (i) number of ribosomal subunits detected / number of predicted ribosomal markers in the MALDI-TOF MS mass range, (ii) total intensity of all peaks per spectra, (iii) fraction of peaks which could be detected in 4 technical replicates. Spectra depicted we acquired using the '25% FA overlay' method and after one day of incubation (3 biological and 4 technical replicates per strain). '****': p-value < 0.0001.

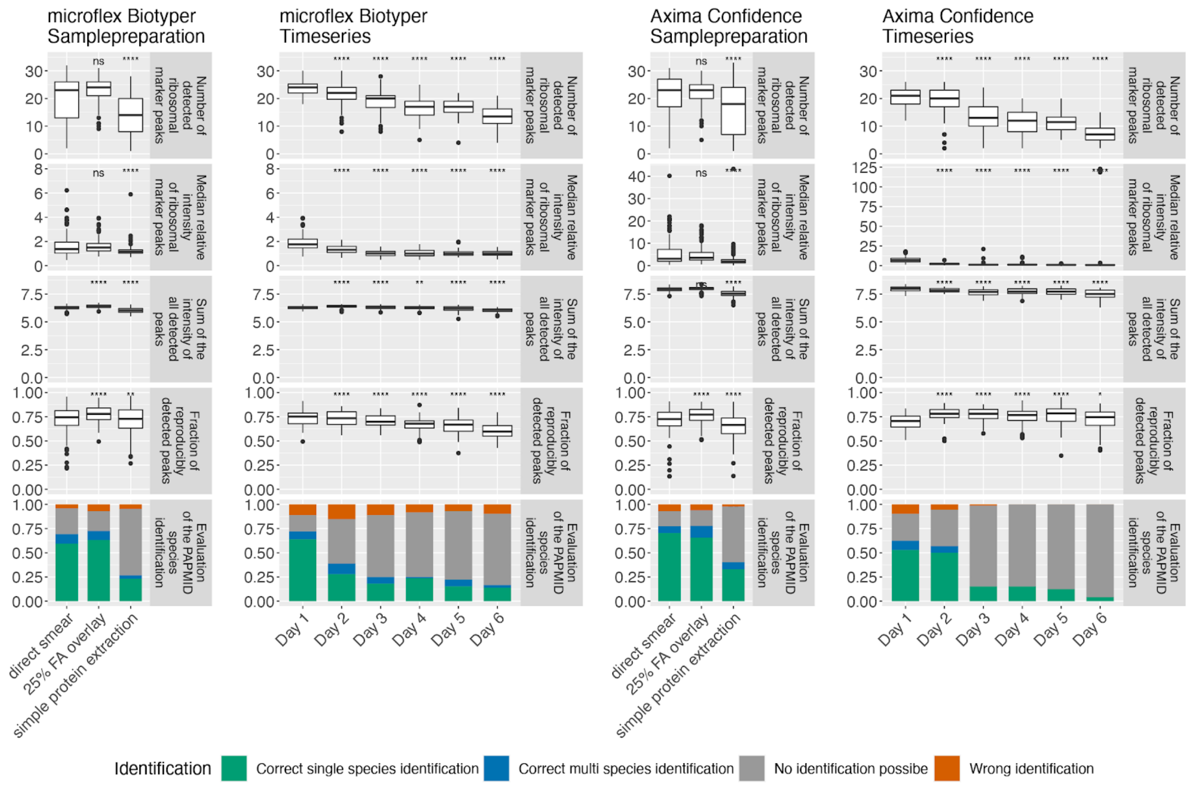

Figure S8 ***Enterobacteriaceae*** (n=18): Comparing different sample preparation protocols (left column) serial dilutions (middle column) and age (right column) of the colony for spectra acquired on a microflex Biotyper and an Axima Confidence MALDI-TOF MS system. Evaluation of a marker based species identification: geen: Correct single species identification; blue: correct multi-species identification; grey: no identification possible: red: wrong species identification.

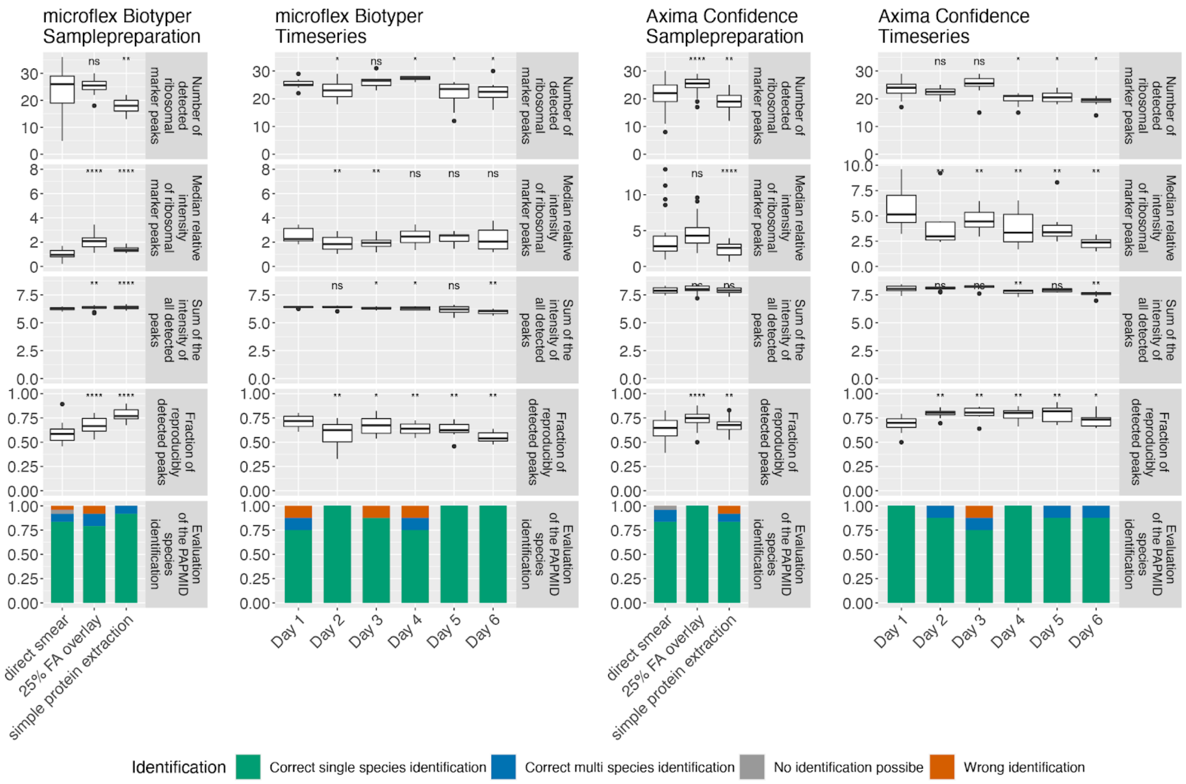

Figure S9 ***Listeria*** (n=2): Comparing different sample preparation protocols (left column) serial dilutions (middle column) and age (right column) of the colony for spectra acquired on a microflex Biotyper and a Axima Confidence MALDI-TOF MS system. Evaluation of a marker based species identification: geen: Correct single species identification; blue: correct multi-species identification; grey: no identification possible: red: wrong species identification.

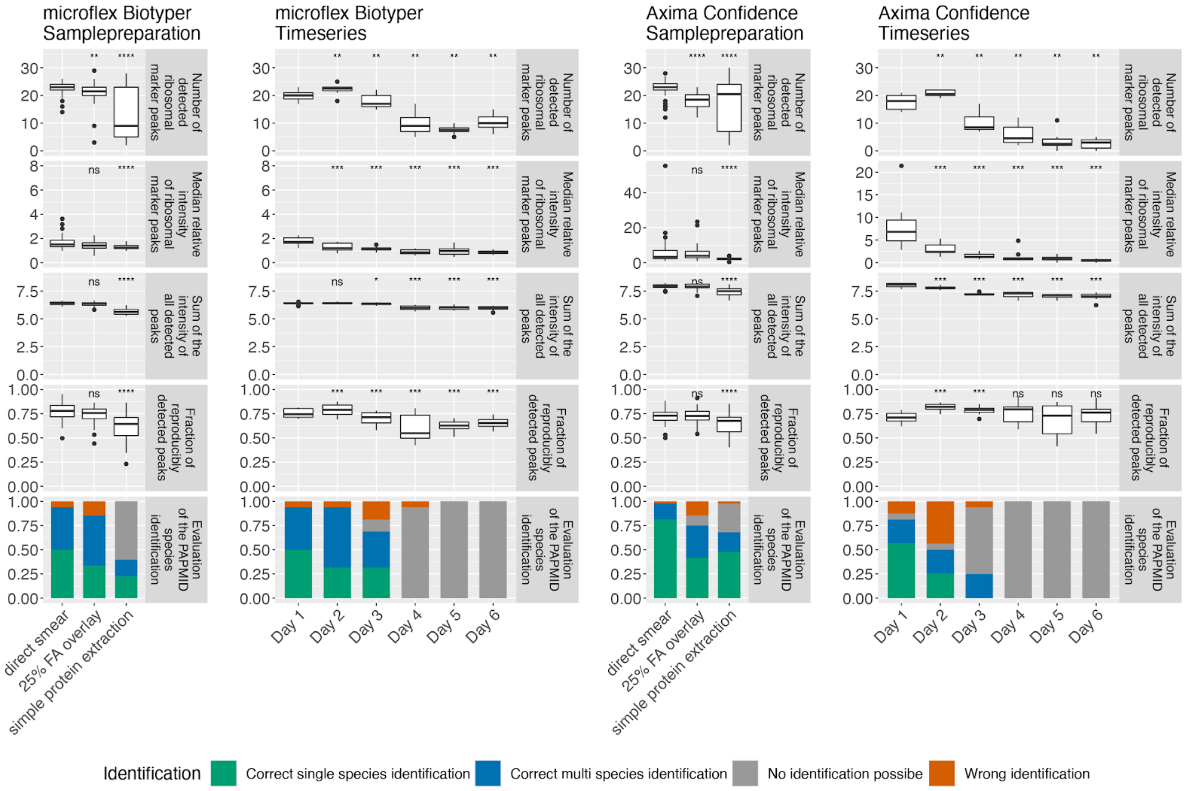

Figure S10 ***Burkholderia*** (n=3): Comparing different sample preparation protocols (left column) serial dilutions (middle column) and age (right column) of the colony for spectra acquired on a microflex Biotyper and a Axima Confidence MALDI-TOF MS system. Evaluation of a marker based species identification: geen: Correct single species identification; blue: correct multi-species identification; grey: no identification possible: red: wrong species identification.

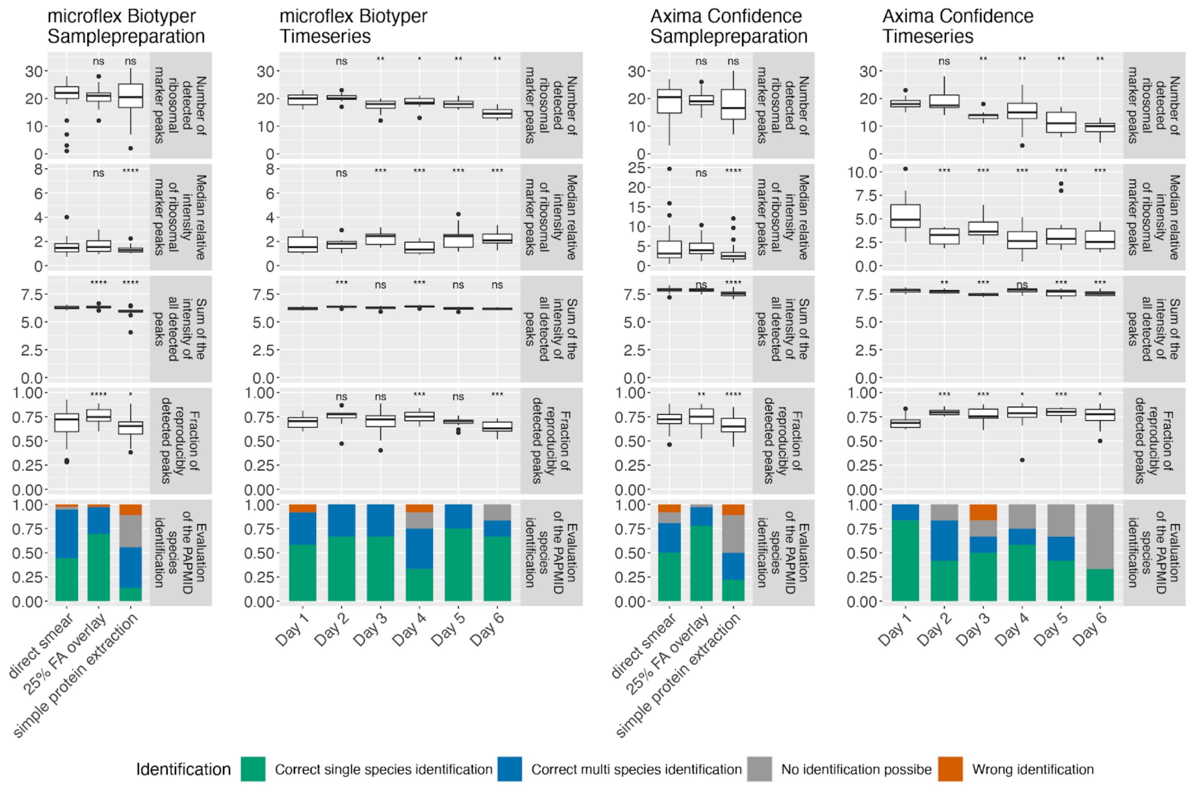

Figure S11 ***Bordetella*** (n=3): Comparing different sample preparation protocols (left column) serial dilutions (middle column) and age (right column) of the colony for spectra acquired on a microflex Biotyper and a Axima Confidence MALDI-TOF MS system. Evaluation of a marker based species identification: geen: Correct single species identification; blue: correct multi-species identification; grey: no identification possible: red: wrong species identification.

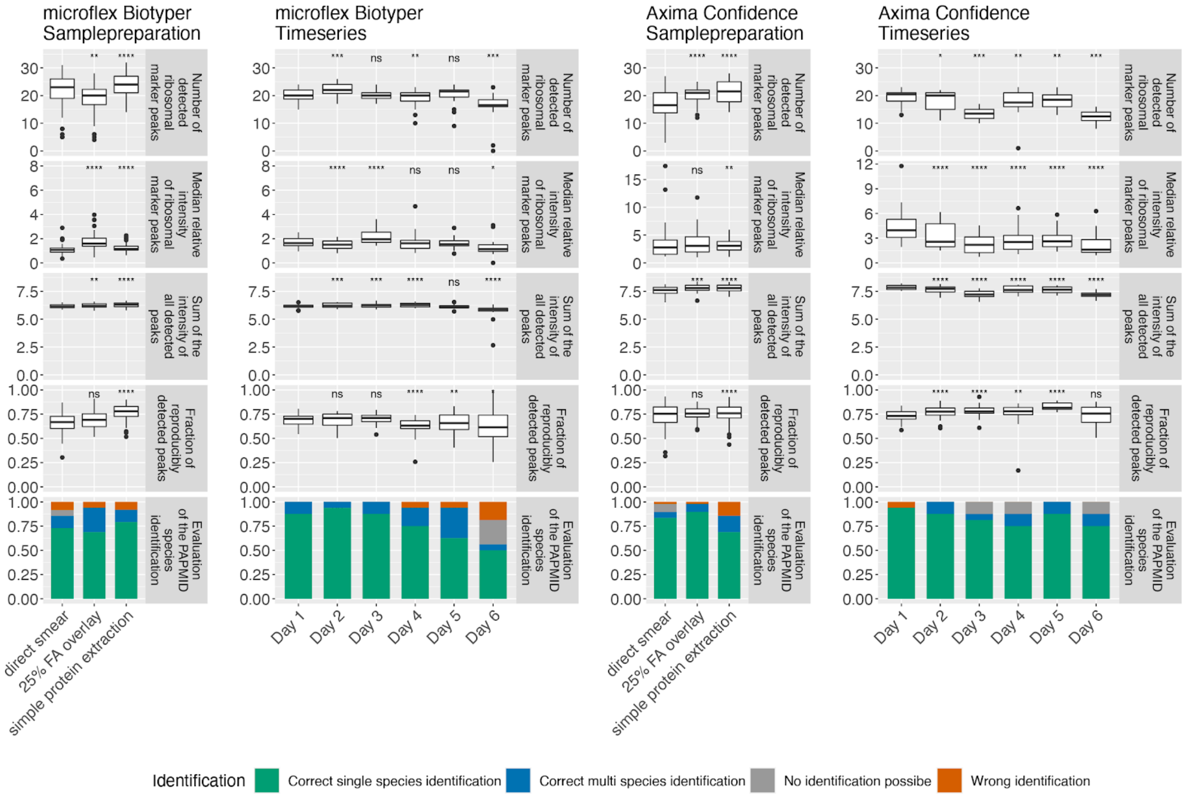

Figure S12 Viridans streptococci (n=3): Comparing different sample preparation protocols (left column) serial dilutions (middle column) and age (right column) of the colony for spectra acquired on a microflex Biotyper and a Axima Confidence MALDI-TOF MS system. Evaluation of a marker based species identification: geen: Correct single species identification; blue: correct multi-species identification; grey: no identification possible: red: wrong species identification.

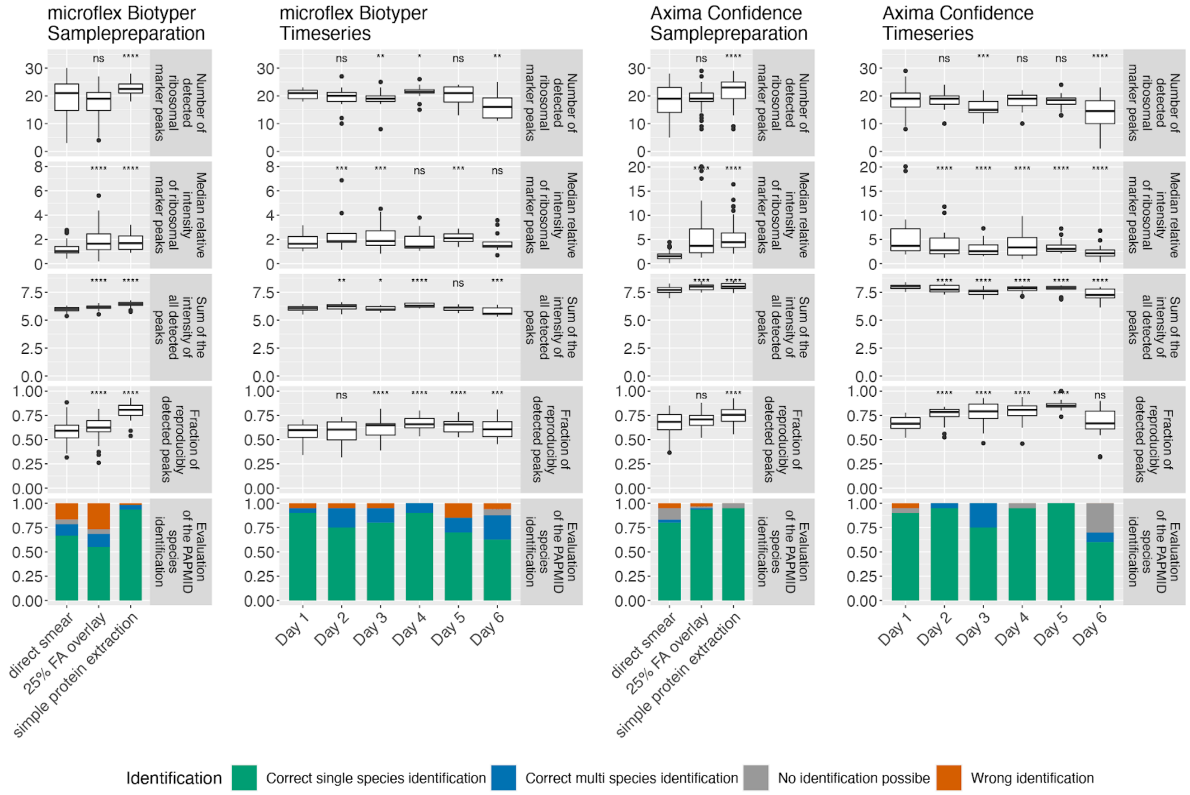

Figure S13 Other *Streptococci* (n=6): Comparing different sample preparation protocols (left column) serial dilutions (middle column) and age (right column) of the colony for spectra acquired on a microflex Biotyper and a Axima Confidence MALDI-TOF MS system. Evaluation of a marker based species identification: geen: Correct single species identification; blue: correct multi-species identification; grey: no identification possible: red: wrong species identification.

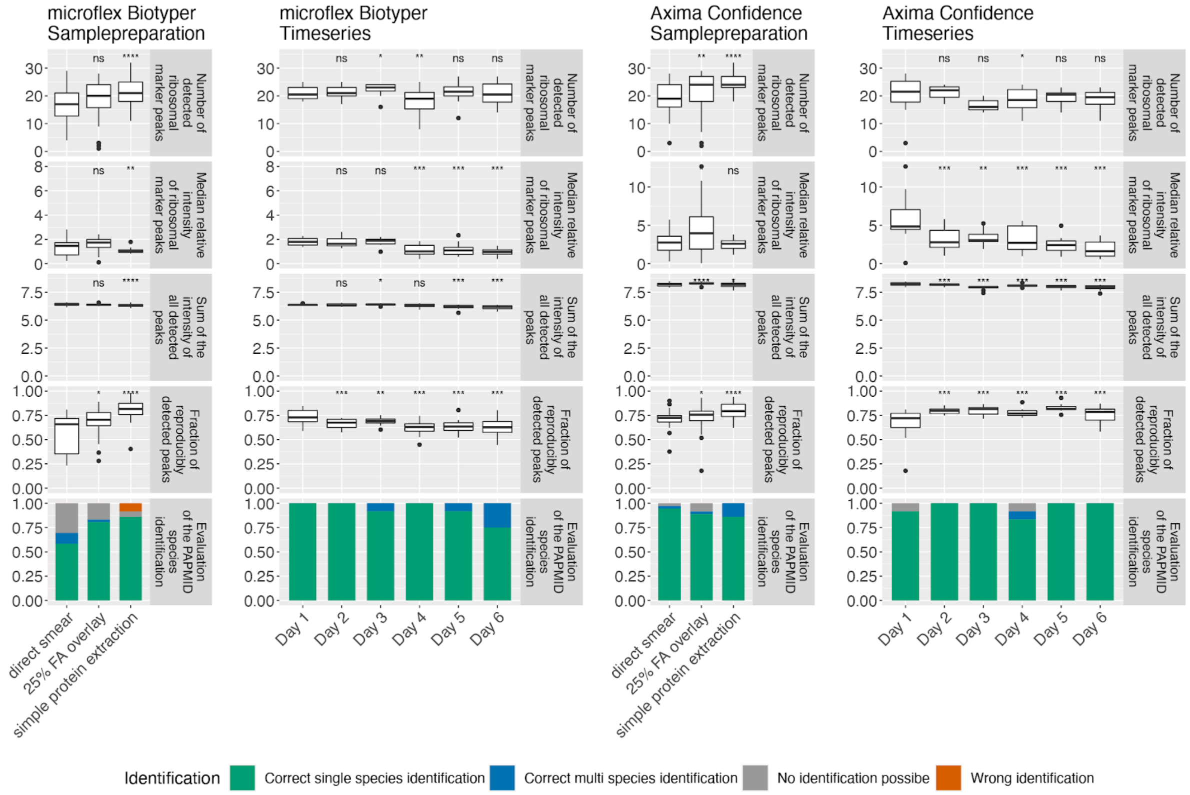

Figure S14 ***Staphylococcus*** (n=3): Comparing different sample preparation protocols (left column) serial dilutions (middle column) and age (right column) of the colony for spectra acquired on a microflex Biotyper and a Axima Confidence MALDI-TOF MS system. Evaluation of a marker based species identification: geen: Correct single species identification; blue: correct multi-species identification; grey: no identification possible: red: wrong species identification.

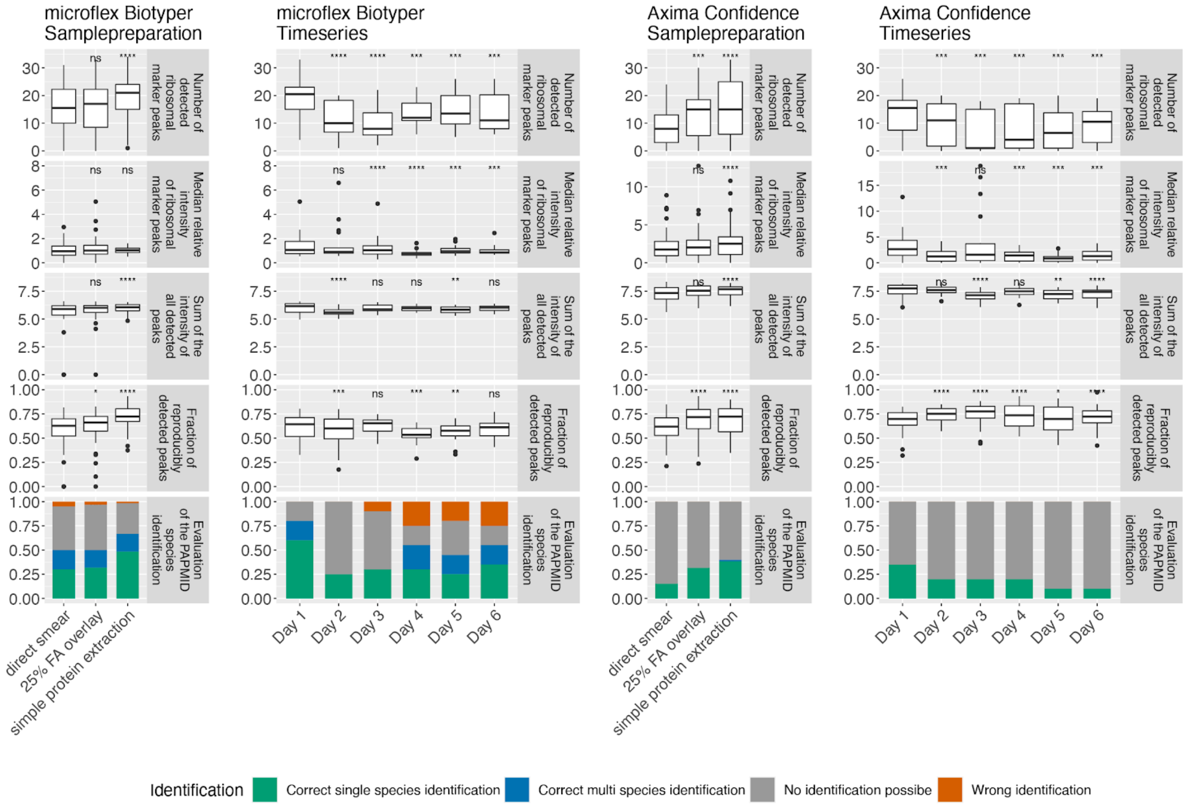

Figure S15 Actinobacteria (n=5): Comparing different sample preparation protocols (left column) serial dilutions (middle column) and age (right column) of the colony for spectra acquired on a microflex Biotyper and a Axima Confidence MALDI-TOF MS system. Evaluation of a marker based species identification: geen: Correct single species identification; blue: correct multi-species identification; grey: no identification possible: red: wrong species identification.

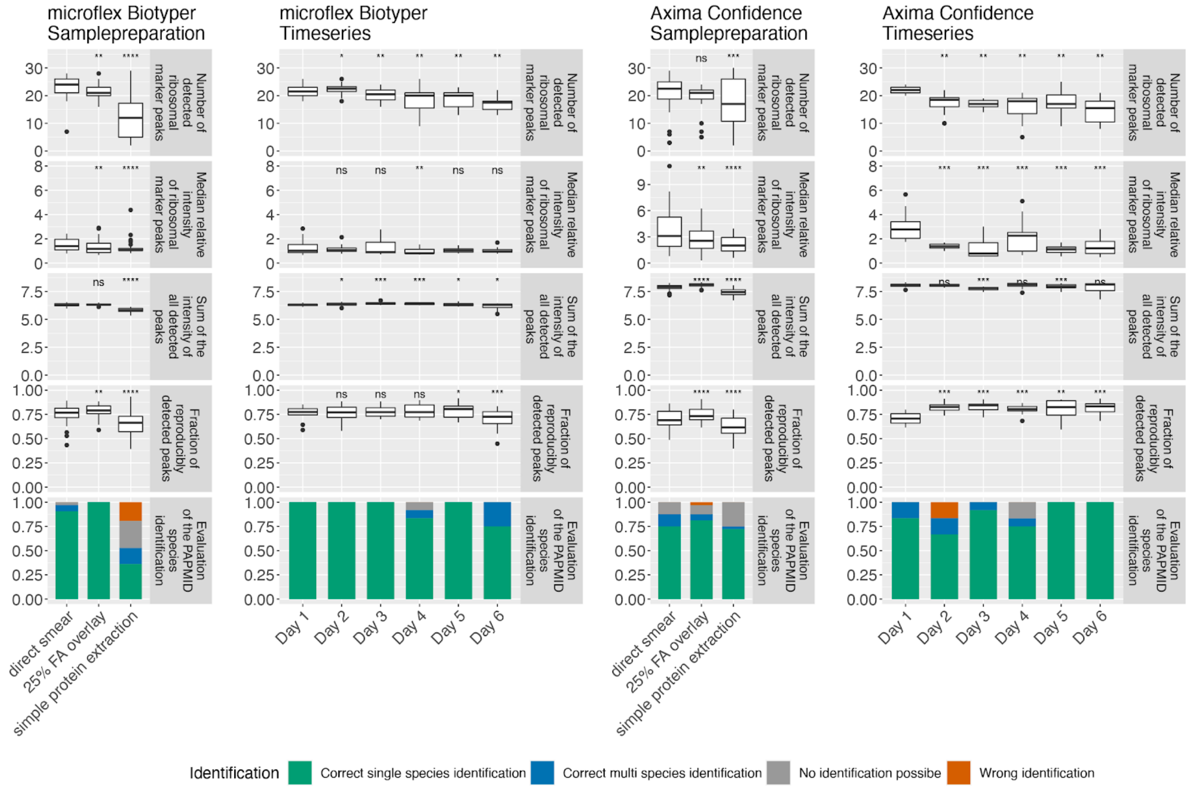

Figure S16 Gram negative Anaerobes (n=3): Comparing different sample preparation protocols (left column) serial dilutions (middle column) and age (right column) of the colony for spectra acquired on a microflex Biotyper and a Axima Confidence MALDI-TOF MS system. Evaluation of a marker based species identification: geen: Correct single species identification; blue: correct multi-species identification; grey: no identification possible: red: wrong species identification.

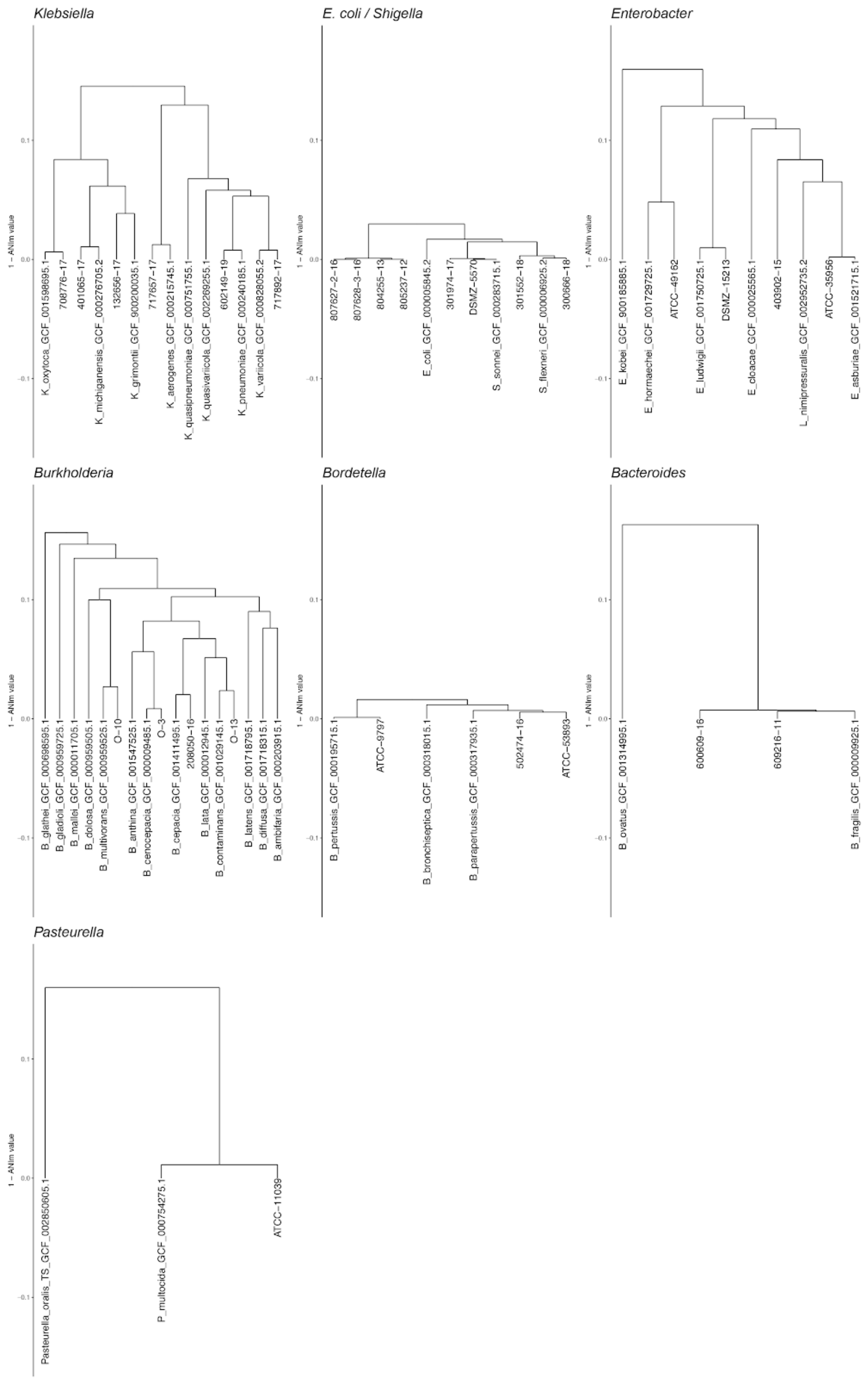

Figure S17: Hierarchical Clustering comparing the ANI of the whole genome assemblies of the gram negative strains included in this study and the type strains of the respective genera.

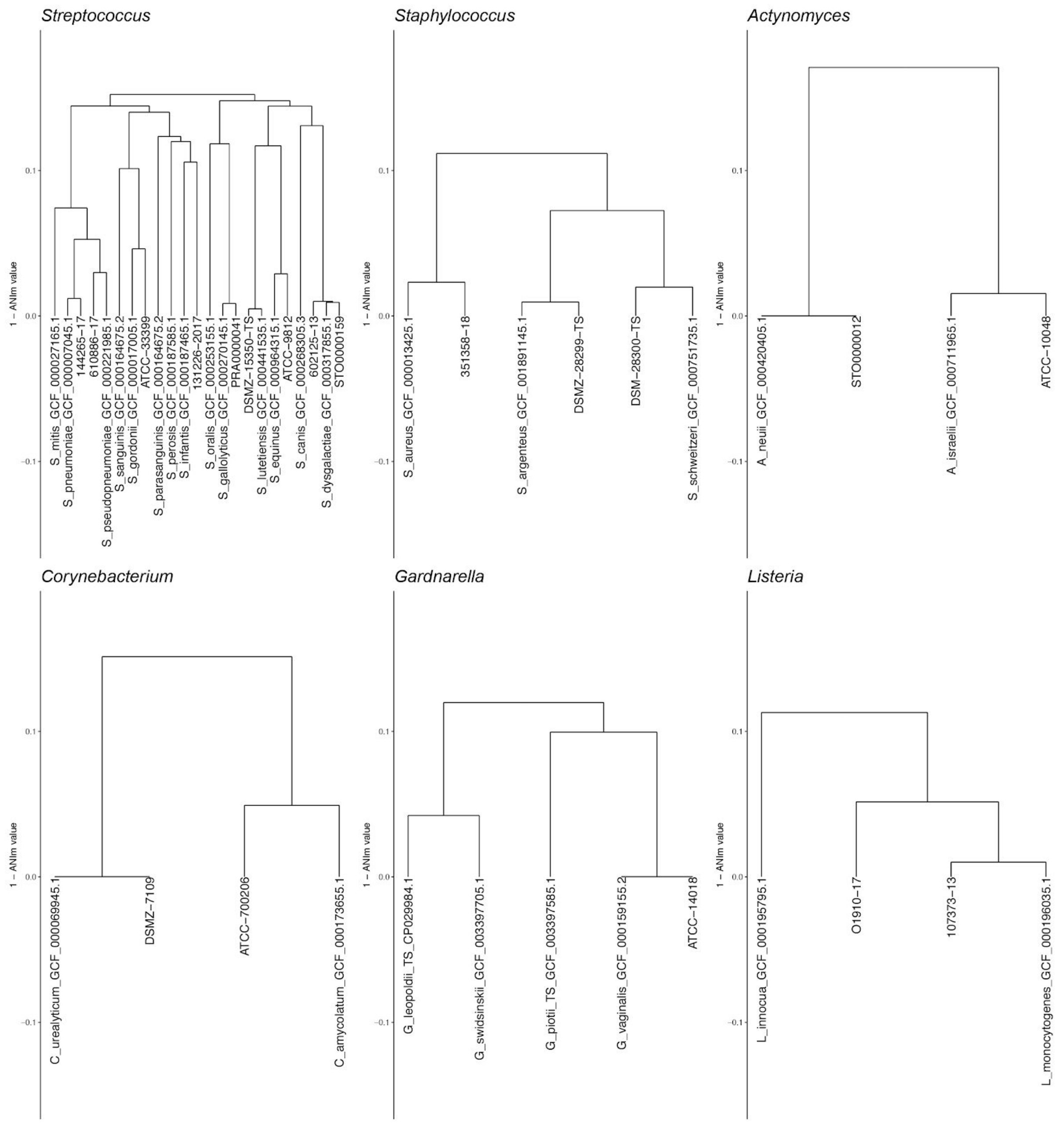

Figure S18: Hierarchical Clustering comparing the ANI of the whole genome assemblies of the gram positive strains included in this study and the type strains of the respective genera.

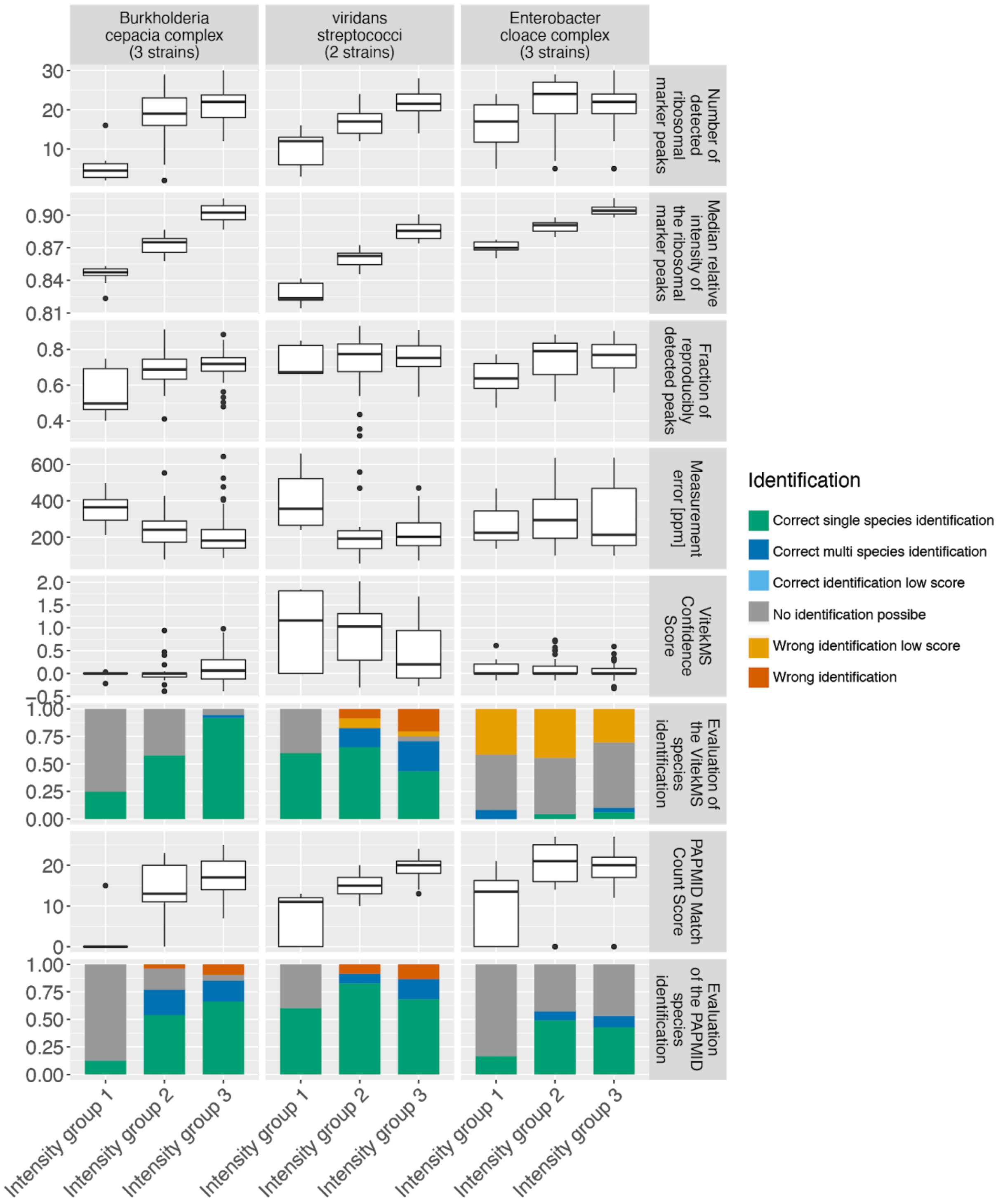

Figure S19: Spectra quality features and species identification for spectra of the *Enterobacter cloacae complex*, the *Burkholderia cepacia complex* and viridans streptococci and spectra acquired on the Axima Confidence. Color Code: green: correct single species identification; dark blue: correct identification, multiple species above threshold or VitekMS Identification Type ‘Low Discriminatory’; grey: no identification possible, yellow: wrong species identified, VitekMS Identification Type ‘Low Discriminatory’, red: wrong species identified, high security (VitekMS Identification Type ‘Single Choice’ and single species identification using a marker based approach).
